# Design and development of an SDR-based system for real-time detection and characterization of drone RF signatures

**DOI:** 10.1038/s41598-026-48925-1

**Published:** 2026-04-20

**Authors:** Mgannem Saber, Radhoine Aloui, Tijeni Delleji, Bilel Hamdi, Sofien Mhatli, Feten Slimeni, Ignacio LLamas-Garro, Ahmed Siala

**Affiliations:** 1 Science and Technology for Defense Lab (STD) Military Research Center, Aouina Military Base, Taieb Mhiri City, 2045 Tunis, Tunisia; 2 Laboratory of the Communication Systems Sys’Com (LR-99-ES21), National Engineering School of Tunis, University of Tunis El Manar, BP. 37 Le Belvédère, 1002 Tunis, Tunisia; 3 SERCOM-Lab, EPT Université de Carthage, La Marsa, Tunis, 2078 Tunisia; 4 ISI KEF, Université de Jendouba, 5 rue saleh Ayech, 7100 El Kef, Tunisia; 5 Centre Tenologic de Telecommunication de catalunya (CTTC/CERCA), 08860 Castelldefels, Spain

**Keywords:** SDR USRPb210, Faster R-CNN, YOLOv5, UAVs, CNNs, RNNs, GPS ANT, detection and STFT, Engineering, Mathematics and computing

## Abstract

The widespread availability of consumer drones has introduced new challenges related to safety, security, and privacy, as these platforms are increasingly misused in sensitive or restricted areas., existing counter-drone technologies–such as radar, optical tracking, and multi-sensor fusion–offer reliable performance but are often prohibitively expensive and impractical for large-scale civilian deployment. This work presents a low-cost framework for real-time drone detection and classification that leverages the radio frequency (RF) emissions exchanged between drones and their controllers. The system is built on a software-defined radio (SDR) platform (USRP B210), which captures RF signals and converts them into spectrograms for analysis using deep learning. A labeled dataset of drone and non-drone signals was developed to train and evaluate detection models. Two state-of-the-art architectures, YOLOv5 and Faster R-CNN, were adapted to this task, with evaluation under varying signal-to-noise ratio (SNR) conditions. Results demonstrate that the proposed system achieves high detection accuracy and robustness even in noisy environments, highlighting its potential as a scalable and practical solution for RF-based drone monitoring.

## Introduction

Unmanned Aerial Systems (UAS) and Unmanned Aerial Vehicles (UAVs), commonly referred to as drones, have experienced a rapid rise in deployment across civilian, commercial, and military domains. They are increasingly used in agriculture, logistics, infrastructure monitoring, surveillance, and recreation due to their low cost, operational flexibility, and reduced dependence on human intervention^1–3^. Their versatility has driven significant innovation, but it has also introduced critical challenges in airspace safety, data security, and privacy protection^4,5^.

The uncontrolled proliferation of drones has resulted in incidents of airspace violations, smuggling, espionage, and even potential terrorist threats^6^. For example, the U.S. Federal Aviation Administration (FAA) regularly reports drone-related incursions near airports, underlining the urgent need for effective counter-drone technologies^4^. Consequently, research on drone detection and identification has expanded rapidly, encompassing radar-based, optical, acoustic, and radio frequency (RF)-based sensing techniques.

Radar-based approaches were among the earliest solutions, leveraging electromagnetic backscatter to identify airborne objects^7^. However, drones’ small radar cross-sections and low-altitude trajectories limit radar effectiveness, particularly in cluttered environments. Enhanced micro-Doppler radar methods have improved performance but remain costly and infrastructure-heavy^8^. Optical and thermal imaging methods, meanwhile, employ visual and infrared sensors combined with computer vision algorithms such as CNNs for object detection^9,10^. These solutions achieve high accuracy under controlled conditions but are constrained by lighting, weather, and line-of-sight limitations.

In recent years, RF-based detection has gained prominence for its ability to passively monitor drone activity using the communication signals exchanged between drones and their controllers^5,6^. Drones continuously emit control, telemetry, and video transmission signals that produce unique spectral and temporal characteristics. Machine learning techniques–especially convolutional neural networks (CNNs) and recurrent neural networks (RNNs)–have demonstrated strong capabilities in extracting these signatures from RF spectrograms^2,3^. Al-Sa’d et al. introduced the DroneRF dataset, achieving 99.7% binary detection accuracy using deep neural networks^5^, while Allahham et al. extended the work to multi-class classification tasks^6^. Other studies applied advanced feature extraction techniques such as Mel-Frequency Cepstral Coefficients (MFCCs), Power Spectral Density (PSD), and Linear Frequency Cepstral Coefficients (LFCCs) to enhance recognition under noisy conditions^7,9,10^.

Unlike traditional protocol-driven receiver designs that require detailed knowledge of waveform structures, modulation schemes, and communication protocols to demodulate and decode a signal, the proposed framework focuses entirely on data-driven RF signature analysis. Conventional receivers often fail or require constant reverse-engineering when faced with proprietary drone communication links (such as frequency-hopping spread spectrum schemes) or encrypted telemetry payloads. In contrast, our data-driven approach treats RF emissions as distinct physical-layer patterns in the time-frequency domain. By analyzing spectro-temporal characteristics–such as burst duration, frequency hopping behavior, and spectral occupancy–the system bypasses the need for bit-level decoding. This allows the framework to reliably detect and classify drone-related RF activity even when communication protocols are proprietary, partially unknown, or constantly evolving. Furthermore, this method maintains robust detection capabilities in congested RF environments and low-SNR conditions, where interference from other wireless technologies would otherwise disrupt standard demodulation and decoding processes.

Despite significant progress, many existing methods rely on complex setups or perform only under controlled laboratory environments. To address these challenges, this paper proposes a low-cost, real-time RF-based drone detection and classification system built on a software-defined radio (SDR) platform (USRP B210). The system captures live RF emissions from drones, converts them into spectrograms, and applies deep learning models (YOLOv5 and Faster R-CNN) to identify drone activity. A labeled dataset of drone and non-drone RF signals was constructed to train and evaluate these models under various signal-to-noise ratio (SNR) conditions.

The main contributions of this work are summarized as follows:

1. The design and implementation of a practical SDR-based architecture for real-time drone RF detection.
2. The creation of a labeled RF dataset to support further research in drone recognition.
3. The adaptation and evaluation of state-of-the-art deep learning architectures (YOLOv5 and Faster R-CNN) for classification under noisy and dynamic environments.

Finally, the remainder of this paper is organized as follows. Section "Drone controlling scenario" introduces the drone control and communication scenario, providing an overview of the RF link structure between drones and controllers. Section "Related work" reviews the existing literature on radar-, optical-, acoustic-, and RF-based detection techniques. Section "Materials and experimental setup" details the experimental setup and SDR-based data acquisition process, while Sect. "Data preparation" describes the dataset generation and annotation procedures. Section "Methods" presents the deep learning architectures and evaluation methodology. The obtained results and validation are discussed in Sect. "Results and validation", followed by an in-depth discussion in Sect. "Discussion". Finally, Sect. "Conclusion" concludes the paper and outlines directions for future research.

## Drone controlling scenario

Most commercial drones rely on a similar control architecture to carry out flight missions. In practice, the same class of communication protocols is typically employed, involving two categories of radio-frequency (RF) links: the uplink and the downlink. The uplink carries command and control instructions transmitted from the remote controller to the drone, while the downlink delivers telemetry data and, in many cases, video streams back to the controller.

**Fig. 1:**
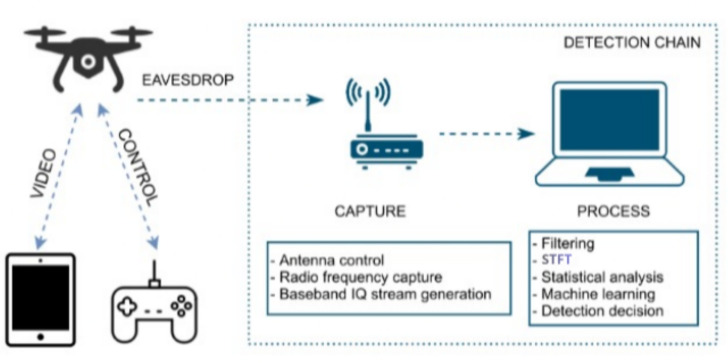
Recording setup (placeholder).

These RF signals are often produced using comparable circuit designs and rely on similar modulation and demodulation techniques^11^. Furthermore, the majority of drones operate in the unlicensed 2.4 GHz and 5 GHz frequency bands^12,13^. Detection systems can therefore exploit passive monitoring strategies, which continuously listen to these communications to capture unique RF signatures that characterize specific drones and their flight modes. Such fingerprints form the basis for a range of recent research efforts aimed at drone detection, identification, and even tracking, as illustrated in Fig. 1.

## Related work

The increasing presence of drones in civilian, commercial, and military contexts has motivated a broad spectrum of detection technologies. Early research focused on conventional radar-based solutions, which identify objects through electromagnetic backscatter. While effective for large targets, radar struggles with drones due to their small radar cross-sections and low-altitude, slow-motion flight patterns. Enhanced approaches using micro-Doppler signatures have been explored, but cost and complexity limit their widespread use^14^.

Non-radar alternatives have therefore gained traction. Imagery-based detection employs electro-optical and thermal sensors combined with computer vision. For example, Thai et al.^15^ extracted motion patterns with convolutional neural networks (CNNs), achieving 93% accuracy, while Schumann et al.^16^ expanded training sets with diverse images to improve drone-versus-bird classification. Saqib et al.^17^ benchmarked CNN models and found Faster R-CNNs more effective, and Aker and Kalkan^18^ demonstrated reliable detection using background-subtracted imagery. More recently, Coluccia et al.^19^ reviewed results from the Drone vs. Bird competition, highlighting progress as well as challenges such as long-range tracking and camera instability. Thermal imaging has also shown promise, particularly for detecting fuel-powered UAVs, which emit stronger heat signatures compared to electric drones^20–22^.

Another line of research explores RF-based detection, leveraging the unique communication signals exchanged between drones and controllers. Al-Sa’d et al.^23^ introduced the DroneRF dataset and trained deep neural networks to achieve 99.7% binary detection accuracy. Building on this, Allahham et al.^24^ applied 1D CNNs for multi-class identification, while Medaiyese et al.^25^ employed XGBoost and wavelet-based features to reach high classification accuracy under different conditions. Other works have utilized spectral features such as MFCCs, PSD, and LFCCs within SVM frameworks, demonstrating robustness in type recognition and mode detection^26^. Nguyen et al.^27^ proposed Matthan, a system that identifies drone presence through Wi-Fi signal fluctuations caused by physical motion, achieving above 90% accuracy across scenarios. Semi-supervised and wavelet-based methods have also been tested, further improving detection in noisy RF environments^28,29^.

Audio-based classification provides another complementary approach by exploiting the distinct acoustic signatures of drone motors and propellers. Bernardini et al.^30^ demonstrated accurate recognition using MFCC features with SVMs, while Jeon et al.^31^ showed recurrent neural networks outperform CNNs and GMMs for real-life sound detection. More recent studies have applied CNNs, RNNs, and hybrid CRNNs to spectrogram features, achieving accuracies above 90%^32–34^. Salman et al.^35^ and Katta et al.^36^ benchmarked multiple deep learning models, finding that GTCC-based features and CNN/LSTM architectures consistently provided strong results, even under noisy conditions.

Overall, while radar, acoustic, and optical modalities offer valuable insights, RF-based approaches stand out for their passive operation, scalability, and ability to function in cluttered environments. When combined with deep learning, RF detection has proven to be a particularly powerful method for real-time drone monitoring and classification.

## Materials and experimental setup

**Fig. 2:**
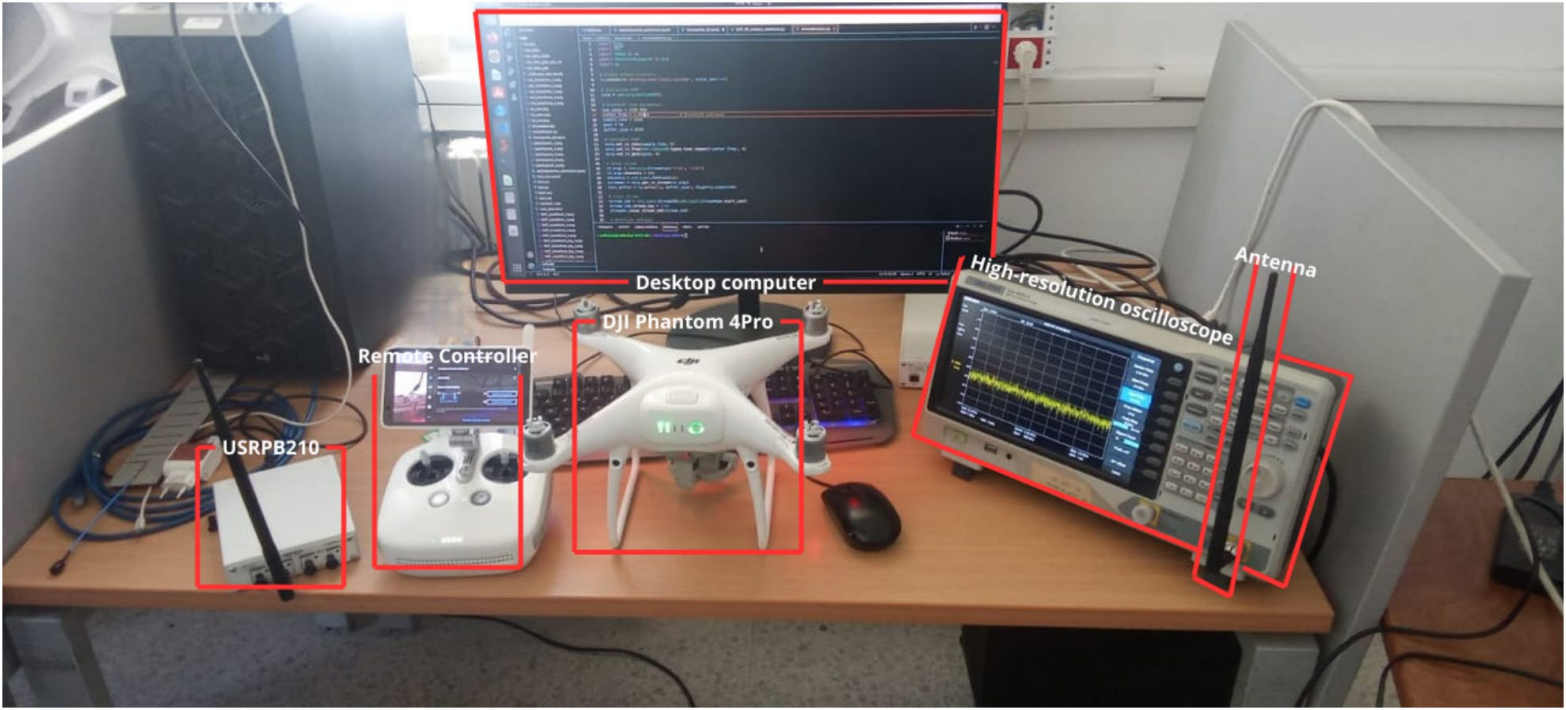
Experimental setup showing the main equipment: desktop computer, DJI Phantom 4 Pro drone, remote controller, USRP B210 SDR, antenna, and high-resolution oscilloscope.

To develop and evaluate the proposed SDR-based drone detection system, several hardware components were employed. The experimental setup is illustrated in Fig. 2, which shows the interconnection of the main equipment.

- *Desktop computer* – served as the central processing unit to control the SDR, acquire data, and execute signal processing and deep learning algorithms. Configured with Ubuntu Linux and Python libraries for real-time spectrogram generation and inference.
- *Software Defined Radio (USRP B210)* – provided wide frequency coverage (70 MHz–6 GHz) and a high sampling rate (up to 61.44 MS/s), enabling reliable capture of drone communication signals in the 2.4 GHz and 5 GHz ISM bands.
- *Antenna system* – omnidirectional antennas connected to the USRP B210 to capture wireless emissions in real time.
- *Drone platform* – a DJI Phantom 4 Pro quadcopter used as the target UAV, operating in the 2.4 GHz band.
- *High-resolution oscilloscope* – integrated to monitor the spectral characteristics of the captured signals and verify frequency occupancy and power levels.

### Software defined radio: Ettus USRP B210

**Fig. 3:**
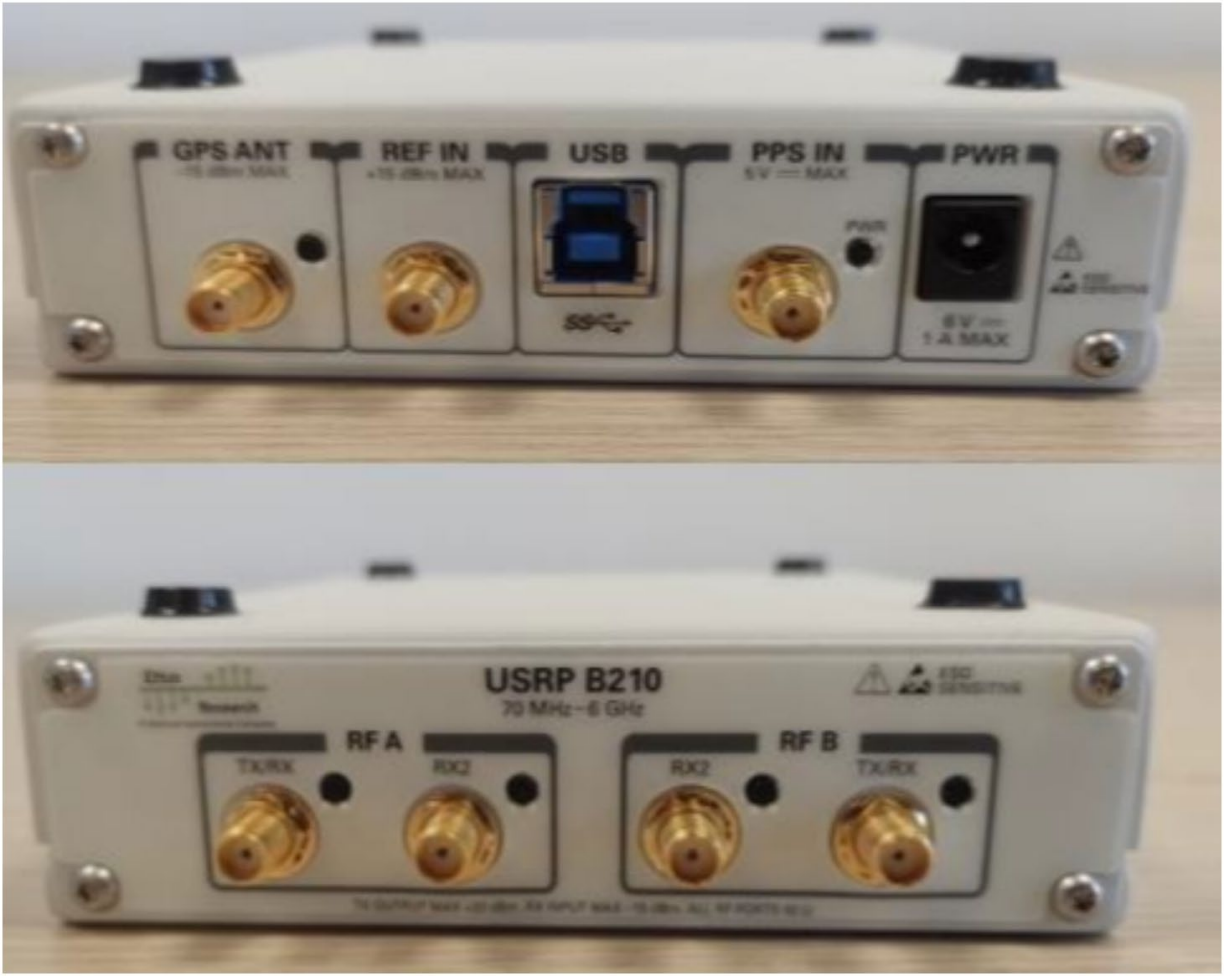
Ettus research USRP B210 software-defined radio used for RF signal acquisition. Front (top) and rear (bottom) panels showing the main input/output interfaces: GPS ANT, REF IN, PPS IN, USB 3.0, PWR, RF A/B TX/RX, and RX2 ports.

The main acquisition hardware employed in this work is the *Ettus Research USRP B210*, a versatile software-defined radio (SDR) platform that offers full-duplex operation with wideband coverage from 70 MHz to 6 GHz^37^. Figure 3 shows the front and rear panels of the device, illustrating its main input and output connectors. The B210 integrates the Analog Devices *AD9361* RF transceiver^38^, which provides dual-channel 12-bit analog-to-digital and digital-to-analog conversion with sampling rates up to 61.44 MS/s. This makes it particularly suited for capturing high-frequency communication signals from drones operating in the 2.4 GHz and 5 GHz ISM bands.

The USRP B210 connects to a host computer through a high-speed *USB 3.0 interface*, supporting real-time signal streaming and control. In this study, it was interfaced with a Linux workstation running GNU Radio and custom Python routines for IQ data capture and spectrogram generation.

*Front-panel interfaces (inputs and control)*

- *GPS ANT* – SMA female connector used for an external GPS antenna. Provides time and location synchronization for distributed or geolocated SDR applications.
- *REF IN (10 MHz)* – accepts a precise 10 MHz reference clock (up to +15 dBm) from an external frequency standard, ensuring coherent frequency alignment across multiple USRP units.
- *PPS IN* – input for a 1 PPS (Pulse Per Second) timing signal (5 V TTL logic). Enables temporal synchronization across SDRs for multi-receiver operation or MIMO experiments.
- *USB 3.0 Port* – serves as the main high-throughput data and control link between the USRP and the host PC.
- *PWR (6 V DC)* – external power supply input rated for 6 V / 3 A max. A power LED indicates proper device startup.

*Rear-panel RF ports (signal I/O)*

- *RF A* and *RF B* – two independent full-duplex transceiver chains, each including:
  - *TX/RX* – shared transmit/receive SMA connector, typically used for single-antenna (SISO) operation.
  - *RX2* – dedicated receive-only port, enabling dual-antenna or MIMO reception.
  Each port supports frequency tuning from 70 MHz to 6 GHz with adjustable gain from 0 to 76 dB^39^.

*Functional overview* The B210 performs several essential functions for drone-signal analysis:

1. *RF front-end tuning* – selects the target frequency band (e.g. 2.4 GHz) and bandwidth.
2. *Signal digitization* – converts analog RF signals into complex baseband IQ samples using 12-bit ADCs.
3. *Host streaming* – transmits digitized IQ data via USB 3.0 to the host for real-time spectrogram generation and deep-learning inference.
4. *Timing synchronization* – uses external 10 MHz REF and 1 PPS inputs to maintain accurate timing across devices.

This configuration allows the capture of drone command-and-control signals, telemetry, and video transmissions, which are later processed to extract spectral features and train deep learning models^40,41^.

### IQ sampling and signal representation

The SDR digitizes received analog RF signals into in-phase *I* and quadrature *Q* components. The discrete-time baseband signal can be expressed as^42^

1 $$\begin{aligned} x[n] = I[n] + j Q[n], \end{aligned}$$

where $I[n]$ and $Q[n]$ denote the real and imaginary parts, respectively, and $j = \sqrt{-1}$. Sampling at period $T_s = 1/f_s$ converts the continuous-time signal $x(t)$ into the discrete sequence:

2 $$\begin{aligned} x[n] = x(n T_s). \end{aligned}$$

To avoid aliasing, the sampling frequency $f_s$ must satisfy the Nyquist criterion:

3 $$\begin{aligned} f_s \ge 2B, \end{aligned}$$

where $B$ is the bandwidth of interest. In our experiments, a sampling rate of $f_s = 56~\textrm{MHz}$ was used to reliably capture the  20 MHz Wi-Fi channels typically exploited by UAVs. The receiver gain was set to 45 dB to improve sensitivity in low-SNR conditions.

The discrete-time signal energy is defined as

4 $$\begin{aligned} E_x = \sum _{n=-\infty }^{\infty } |x[n]|^2, \end{aligned}$$

and in the presence of additive noise $w[n]$, the received signal is

5 $$\begin{aligned} y[n] = x[n] + w[n]. \end{aligned}$$

The signal-to-noise ratio (SNR) is then given by

6 $$\begin{aligned} \textrm{SNR} = \frac{\sum _n |x[n]|^2}{\sum _n |w[n]|^2}, \quad \textrm{SNR}_\textrm{dB} = 10 \log _{10} \frac{\sum _n |x[n]|^2}{\sum _n |w[n]|^2}. \end{aligned}$$

Autocorrelation and cross-correlation functions are classical tools to analyze signal patterns^43,44^:

7 $$\begin{aligned} R_x[k] = \sum _{n=-\infty }^{\infty } x[n] x^*[n-k], \quad R_{xr}[k] = \sum _{n=-\infty }^{\infty } x[n] r^*[n-k], \end{aligned}$$

where $r[n]$ is a reference signal.

### Time–frequency transformation

To analyze the temporal and spectral characteristics of the captured signals, the Short-Time Fourier Transform (STFT) was applied^45,46^:

8 $$\begin{aligned} X(m,\omega ) = \sum _{n=-\infty }^{\infty } x[n] w[n-m] e^{-j \omega n}, \end{aligned}$$

where $w[n]$ is a sliding window, $m$ indexes time, and $\omega$ denotes angular frequency. The spectrogram, representing signal power in time and frequency, is then,

9 $$\begin{aligned} S(m,\omega ) = |X(m,\omega )|^2. \end{aligned}$$

Parseval’s theorem provides the connection between time- and frequency-domain energy^42^:

10 $$\begin{aligned} \sum _{n=-\infty }^{\infty } |x[n]|^2 = \frac{1}{2\pi } \int _{-\pi }^{\pi } |X(e^{j\omega })|^2 d\omega , \end{aligned}$$

ensuring that the STFT preserves the energy content of the signal.

### Acquisition workflow

**Fig. 4:**
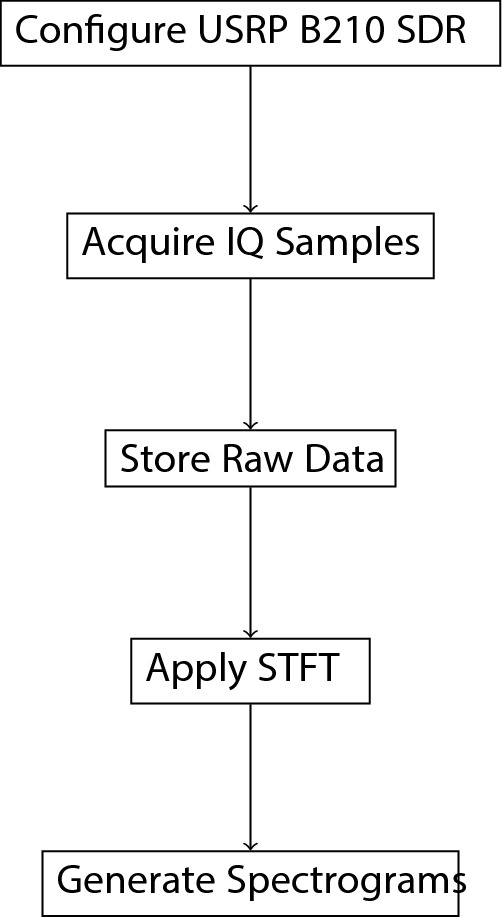
RF data acquisition pipeline.

The acquisition pipeline is summarized in Fig. 4. The process begins with configuring the USRP B210, followed by IQ sampling, storage of raw data, and spectrogram generation through STFT.While quantities such as SNR and autocorrelation were not explicitly computed in our experiments, their theoretical basis supports the validity of the STFT-based spectrograms as a reliable representation of UAV signals in complex RF environments^38,39^. These spectrograms are then used in the next stage for dataset preparation and annotation (see Sect. "Data preparation").

### Illustrative example

**Fig. 5:**
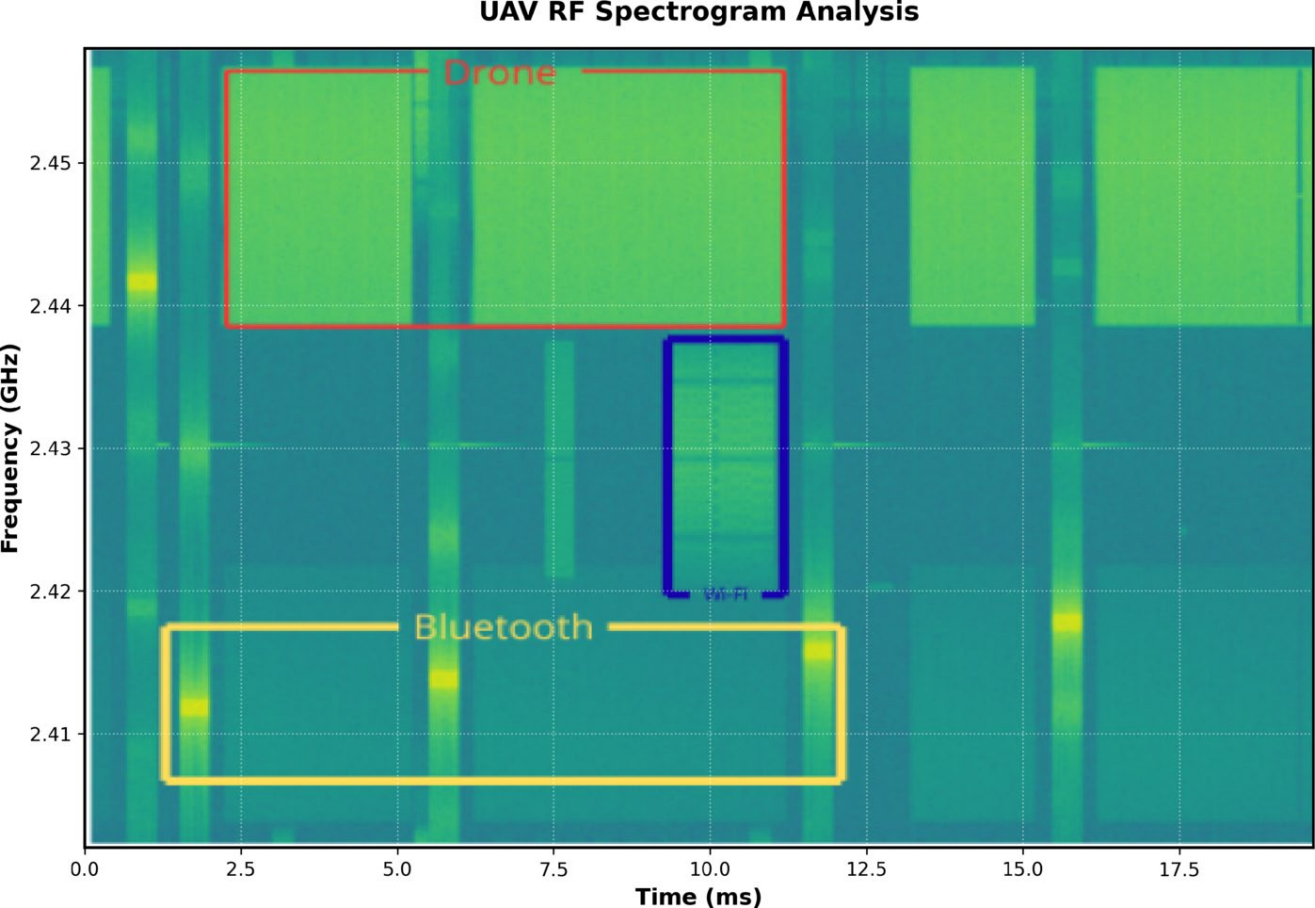
Spectrum captured in the 2.4 GHz ISM band using the USRP B210. Narrowband emissions correspond to drone telemetry/control, wideband channels to Wi-Fi, and short bursts to Bluetooth.

An example spectrum capture is shown in Fig. 5. Distinct signal types coexist in the 2.4 GHz band:drone telemetry and control links, Wi-Fi channels, and Bluetooth frequency-hopping bursts. This coexistence illustrates the difficulty of reliable drone detection in crowded RF environments, motivating the need for robust machine learning models.

During the RF data collection process, the Wi-Fi signals were captured under active data transmission conditions. A laptop was connected to a wireless router and continuous UDP traffic of approximately 20 Mbps was generated to ensure sustained OFDM activity. For Bluetooth signals, the transmissions were captured from a wireless headset actively streaming audio, which resulted in continuous frequency-hopping communication. Therefore, both Wi-Fi and Bluetooth signals used in the dataset correspond to active transmission scenarios rather than idle conditions. To further illustrate the impact of transmission state on the RF signatures, additional spectrogram examples comparing idle and active transmission modes for both Wi-Fi and Bluetooth signals are provided in the supplementary material.

### Transmission-state spectrogram comparison

**Fig. 6:**
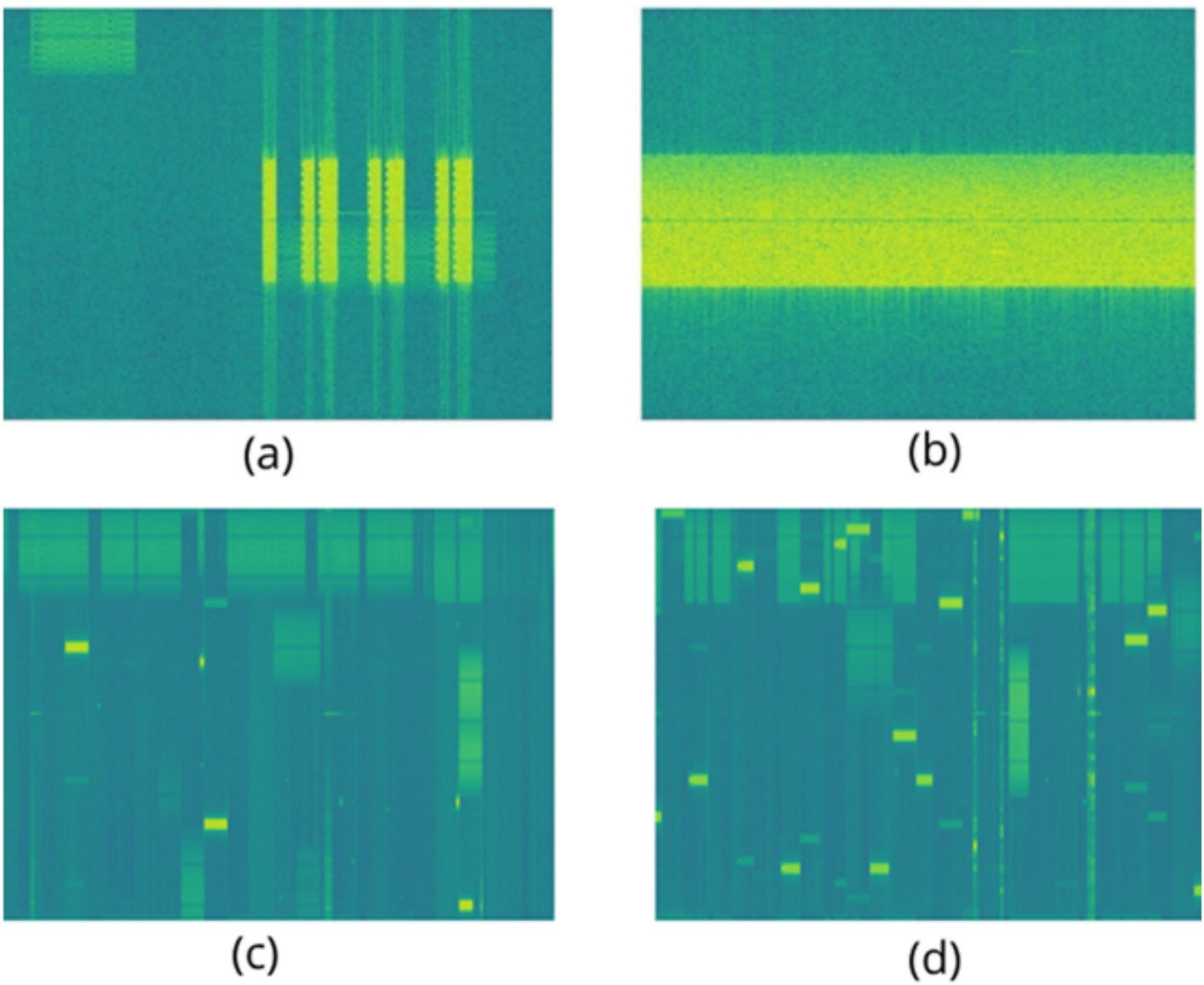
Spectrogram comparison of wireless signals under different transmission conditions: (**a**) Wi-Fi idle state showing sparse beacon-frame activity, (**b**) Wi-Fi active transmission with continuous OFDM packets occupying a 20 MHz channel, (**c**) Bluetooth idle state with occasional control packets, and (**d**) Bluetooth active transmission during audio streaming, exhibiting dense frequency-hopping bursts across the 2.4 GHz band.

**Fig. 7:**
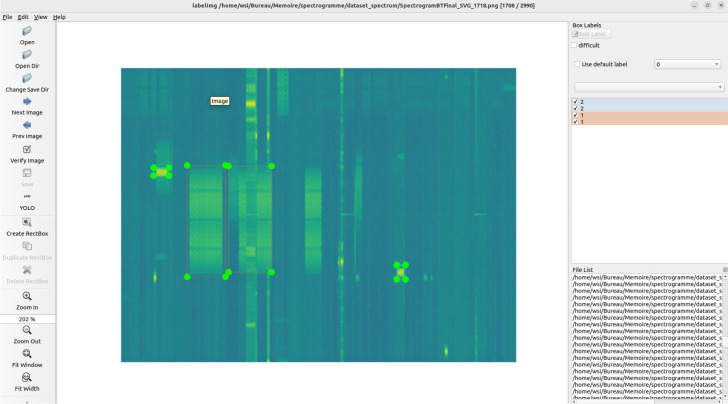
Example of annotated spectrogram using *LabelImg*. Bounding boxes identify Drone, Wi-Fi, and Bluetooth signals.

To better illustrate the impact of transmission activity on signal morphology, additional spectrograms were generated for Wi-Fi and Bluetooth signals under idle and active transmission conditions. These examples help clarify the differences between low-traffic states and realistic communication scenarios. For Wi-Fi signals, the idle condition corresponds to a device connected to a network but not actively transferring data. In this state, the spectrum mainly contains periodic beacon frames, which appear as sparse and intermittent spectral bursts. In contrast, during active data transmission, continuous OFDM-based Wi-Fi packets occupy a full 20 MHz channel, producing dense and sustained spectral activity. Bluetooth signals exhibit a different behavior due to their frequency-hopping spread spectrum (FHSS) modulation. When idle, Bluetooth devices transmit only occasional control packets, resulting in isolated bursts across the hopping channels. During active operation–such as audio streaming through a wireless headset–the hopping pattern becomes much more frequent, creating a dense sequence of narrowband bursts across the band (Table 1). Figure 6 illustrates these differences by presenting representative spectrograms for Wi-Fi and Bluetooth signals in both idle and active transmission modes. These examples demonstrate how transmission load affects the time–frequency structure of the signals and highlight the importance of collecting training data under realistic network traffic conditions when developing machine learning models for drone detection in congested RF environments (Fig. 7).

**Table 1:** Classification and visual characteristics of wireless signal spectrograms under different transmission conditions.

Label	Classification	Visual characteristics
(a)	Wi-Fi idle state	Shows sparse, periodic horizontal bursts. These correspond to beacon frames transmitted by the access point to maintain network connectivity without active data transmission.
(b)	Wi-Fi active state	Shows a dense, continuous, and wide spectral block, representing a fully utilized 20 MHz OFDM channel under approximately 20 Mbps UDP traffic.
(c)	Bluetooth idle state	Displays sparse, isolated narrowband spots across the frequency range, corresponding to occasional control and paging packets used for synchronization.
(d)	Bluetooth active state	Shows a high density of narrowband bursts rapidly hopping across the band, illustrating the FHSS pattern characteristic of active audio streaming.

## Data preparation

Following acquisition, the IQ samples were transformed into spectrograms, which were then annotated to produce a labeled dataset for training deep learning models. A total of *5813 spectrograms* were generated, with *18,984 labeled bounding boxes*.

### Annotation process

Spectrograms were annotated using the *LabelImg* tool. Bounding boxes were drawn around distinct RF patterns and assigned class labels:

- Drone signals – narrowband emissions with stable frequency structures,
- Wi-Fi signals – wideband OFDM channels (20–40 MHz),
- Bluetooth signals – short, frequency-hopping bursts.

### Challenges

- Overlapping signals required precise bounding box placement.
- Low-SNR Bluetooth signals were faint and difficult to annotate.
- Truncated signals at spectrogram edges were annotated conservatively.

### Final dataset

The dataset was split into training and validation subsets, balanced across classes. It wasYOLOv5 consistently outperform later used to train *YOLOv5* and *Faster R-CNN* models, whose performance is discussed in Sect. "Methods".

The dataset is composed of three primary classes based on the type of RF signal:

- Drone signals (Class 0): narrowband emissions with relatively stable frequency structures.
- Wi-Fi signals (Class 1): wideband OFDM channels typically occupying 20–40 MHz bandwidth.
- Bluetooth signals (Class 2): short-duration frequency-hopping bursts across the 2.4 GHz band.

## Methods

### Model architectures

Two complementary object detection models were selected to address the problem of drone signal recognition from spectrograms: *YOLOv5* and *Faster R-CNN*.

YOLOv5, a one-stage detection framework, is designed for high efficiency and real-time inference. It directly predicts bounding boxes and class probabilities from feature maps, making it suitable for time-sensitive applications. In this study, it was adapted to operate on spectrograms derived from RF signals, enabling rapid identification of drone, Wi-Fi, and Bluetooth transmissions.

Faster R-CNN, in contrast, follows a two-stage approach. A Region Proposal Network (RPN) first generates candidate bounding boxes, which are then refined through classification and regression layers. By combining the ResNet-50 backbone with feature pyramid networks (FPN), the architecture achieves robust localization even in challenging low-SNR conditions. While slower, this model is known for high precision, particularly in cases where overlapping signals are present.

### Evaluation strategy

The models were assessed using a common annotated dataset of 5,813 spectrograms. Both architectures were trained on 80% of the dataset, with the remaining 20% reserved for validation and testing.

Performance evaluation relied on several complementary metrics:

- *Accuracy:* The proportion of correctly predicted classes.
- *Precision, recall, and F1-score:* Indicators of detection reliability and robustness against false positives or false negatives.
- *Balanced accuracy:* To account for class imbalance across drone, Wi-Fi, and Bluetooth signals.
- *Inference speed (FPS):* The average number of spectrograms processed per second, reflecting real-time capability.

This evaluation framework was designed to not only compare raw detection accuracy but also highlight trade-offs between speed and robustness, which are critical for real-world drone monitoring systems.

## Results and validation

### Introduction

This section presents the evaluation of the proposed framework using two state-of-the-art object detection models, **YOLOv5** and *Faster R-CNN*, applied to spectrogram-based datasets. Four experiments were designed to compare their performance: baseline detection, signal annotation, localization accuracy, and cross-band generalization. Results are reported with both quantitative metrics (accuracy, precision, recall, F1-score, inference speed) and qualitative analyses (confusion matrices and annotated outputs).

### Experiment 1: baseline comparison

This experiment evaluates the performance of two state-of-the-art object detection models–*YOLOv5* and *Faster R-CNN*–for the task of drone detection using spectrogram images. The dataset used in this study consists of a total of 5,813 spectrogram images. To ensure a fair and consistent comparison, the dataset was divided into two parts: 80% of the images were used for training the models, while the remaining 20% were allocated for validation and testing.

Both models were trained on this dataset under similar conditions, allowing their results to be directly compared. After training, the models were saved for further evaluation, ensuring that the same trained versions could be consistently tested and analyzed. The evaluation was conducted using standard performance metrics, including accuracy, precision, recall, F1-score, and inference speed (FPS). This setup not only provides a direct comparison between YOLOv5 and Faster R-CNN but also highlights their strengths and weaknesses in detecting drones from RF spectrogram representations.

#### Overall metrics

**Table 2:** Overall performance comparison between Faster R-CNN and YOLOv5.

Metric	Faster R-CNN	YOLOv5	Winner & analysis
Accuracy	0.8899	0.9082	YOLOv5 (+2%)
Precision	0.8898	0.9115	YOLOv5 (higher positive prediction quality)
Recall	0.8899	0.9082	YOLOv5 (slightly more complete detection)
F1-score	0.8894	0.9085	YOLOv5 (better balance)
Avg Confidence	N/A	0.8309	N/A

Table 2 highlights the comparative performance of Faster R-CNN and YOLOv5 across common evaluation metrics. The results show that YOLOv5 consistently outperforms Faster R-CNN in accuracy, precision, recall, and F1-score. While the gains in accuracy and recall are modest, they indicate YOLOv5’s ability to detect slightly more objects overall. Its higher precision reflects a stronger capacity to minimize false positives, meaning that its detections are more reliable in practical scenarios. The superior F1-score further confirms YOLOv5’s balanced trade-off between completeness and correctness of detection. Notably, YOLOv5 also provides average confidence scores for its predictions, an additional layer of interpretability that Faster R-CNN lacks. Collectively, these findings suggest that YOLOv5 is the more robust option when both reliability and detection balance are prioritized.

#### Overfitting analysis

**Fig. 8:**
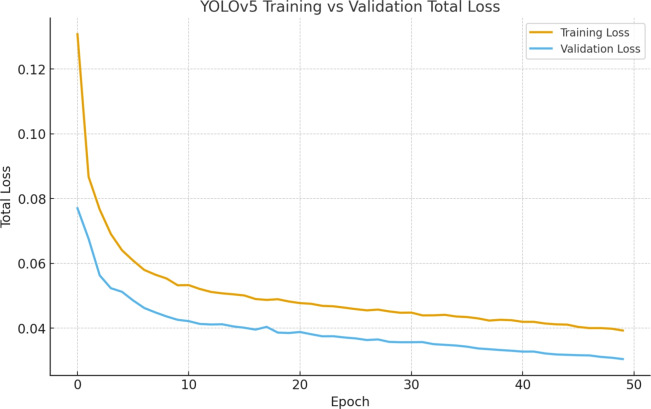
YOLOv5 training and validation total loss evolution. The close convergence indicates no overfitting.

To evaluate whether the models suffer from overfitting, we analyzed both their training and validation performances through precision, recall, confidence-based curves, and loss evolution. Overfitting typically appears when a model performs very well on the training data but fails to generalize on unseen validation or test data – often resulting in high training accuracy but noticeably lower validation precision or recall, along with a significant gap between training and validation loss (Fig. 8).

**Fig. 9:**
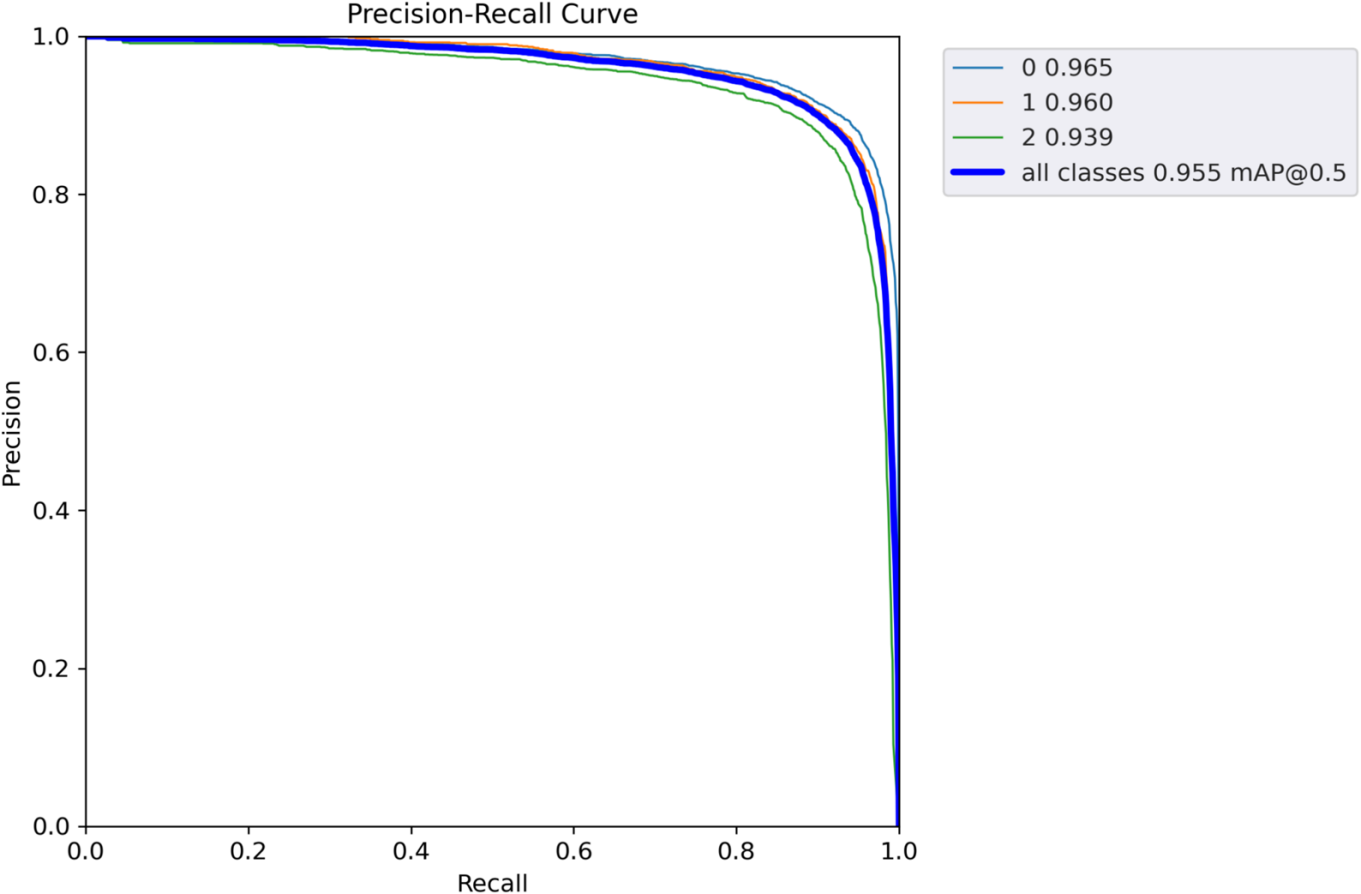
Precision–Recall curve for YOLOv5 indicating stable generalization and high mAP@0.5.

**Fig. 10:**
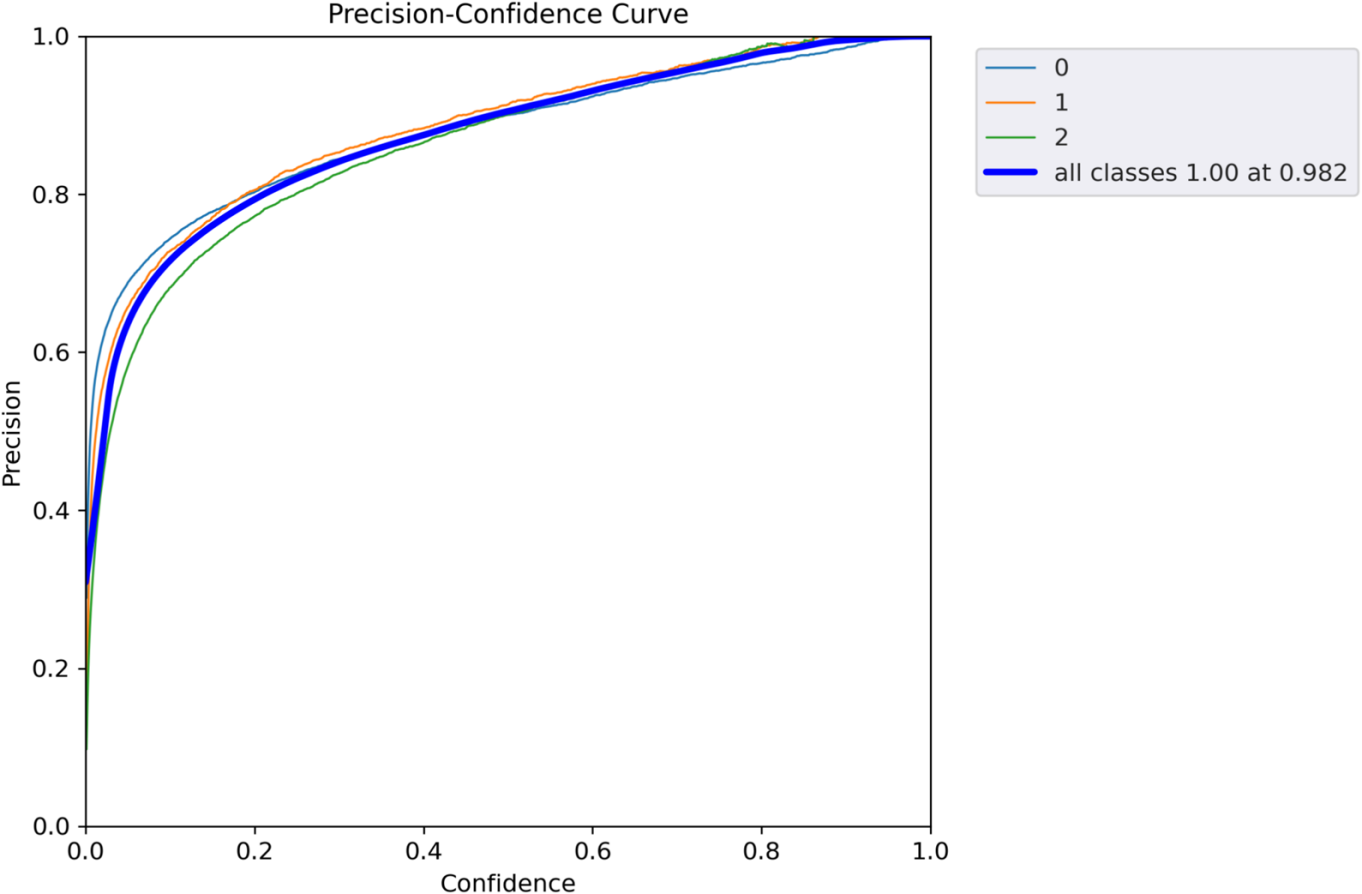
Precision–Confidence curve showing smooth performance across confidence thresholds.

**Fig. 11:**
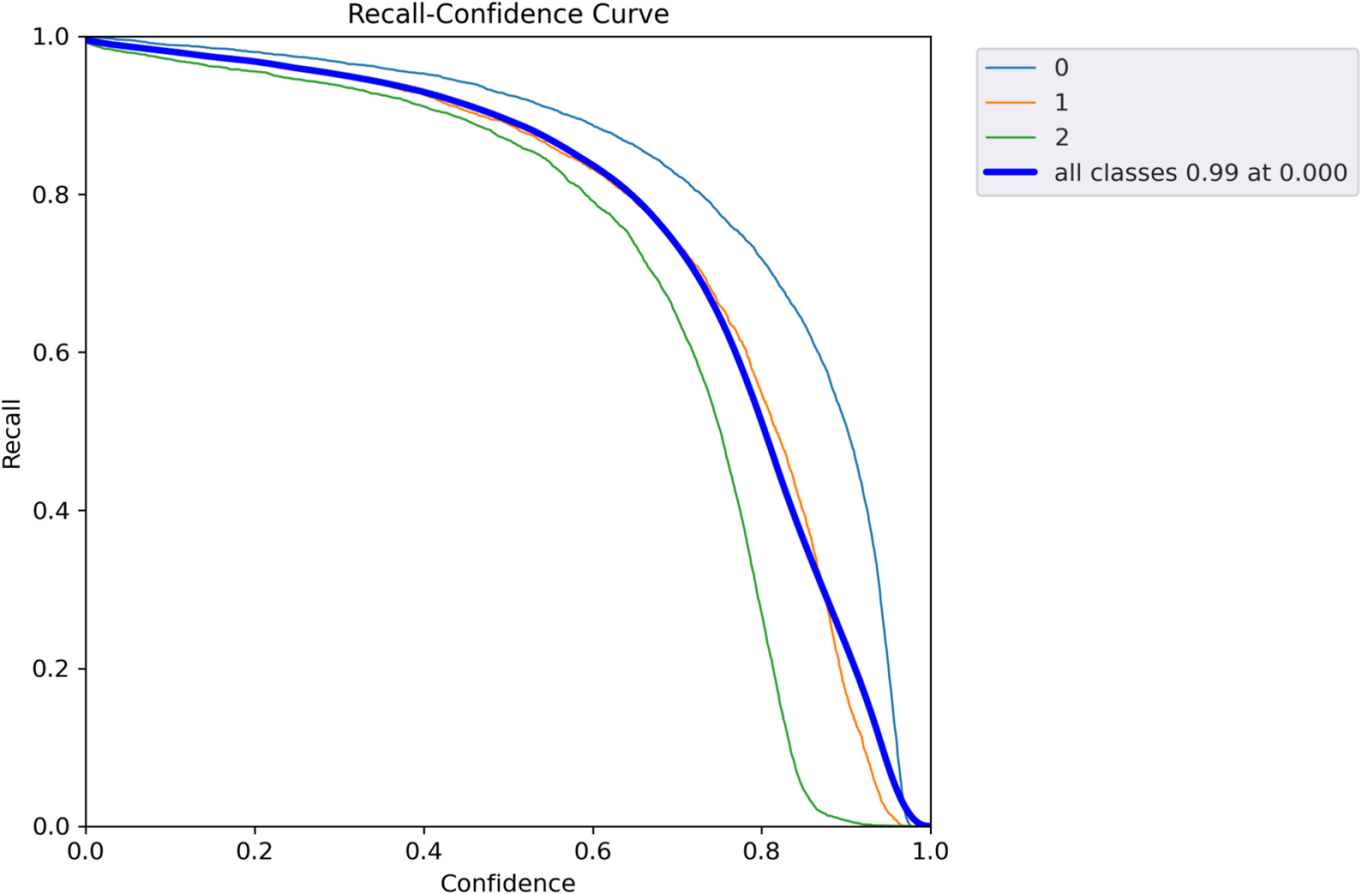
Recall–Confidence curve confirming strong recall stability without overfitting.

For YOLOv5, Figs. 9, 10 and 11 illustrate the model’s Precision–Recall (PR), Precision–Confidence (P–C), and Recall–Confidence (R–C) curves, respectively. These curves show smooth and consistent performance across confidence thresholds, with the PR curve remaining close to the top-right corner – indicating high precision and recall for all classes. The mean Average Precision (mAP@0.5) of 0.955 confirms that YOLOv5 maintains strong generalization across all drone classes.

The P–C and R–C curves show stable precision and recall trends as confidence increases, without any sudden drops or oscillations. This stability suggests that the model’s confidence scores are well-calibrated and not excessively tuned to training data. Furthermore, the loss evolution shown in Fig. 8 demonstrates that both training and validation losses decrease smoothly and converge closely, with no significant divergence – further confirming the absence of overfitting.

**Fig. 12:**
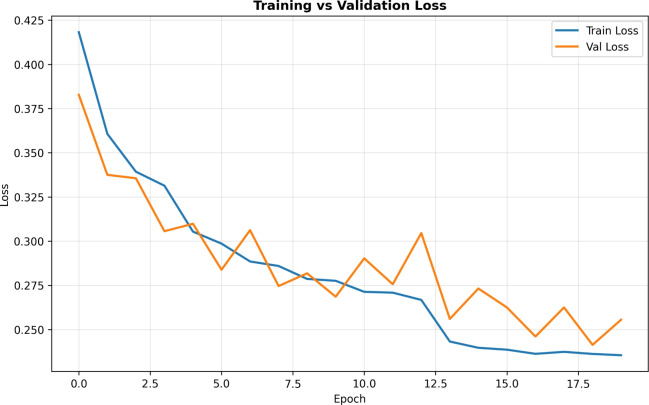
Faster R-CNN training and validation loss curves. Stable parallel decrease suggests no overfitting.

In contrast, Faster R-CNN shows lower precision and recall overall, but this reduction is consistent across both training and validation sets, indicating that the model underfits slightly rather than overfits. Its loss curves in Fig. 12 reveal parallel decreasing trends for training and validation losses, with no widening gap, which supports the conclusion that overfitting is not present. Its lower performance, especially on Classes 1 and 2, appears to result from difficulty generalizing complex signal variations rather than memorizing the training data.

Overall, based on both quantitative metrics, loss evolution, and curve analysis, there is no significant evidence of overfitting in YOLOv5 or Faster R-CNN. Both models maintain consistent loss, precision, recall, and confidence distributions across validation data, suggesting effective generalization. Faster R-CNN, while less accurate, also does not show typical overfitting behavior but instead demonstrates limited representational power.

#### Inference speed

Table 3 highlights the computational gap between the two models. Faster R-CNN processes each frame in nearly 688 ms (about 1.5 FPS), which is unsuitable for real-time use. In contrast, YOLOv5 achieves an average of 37 ms per frame, exceeding 27 FPS. This makes YOLOv5 more than 18 times faster and directly applicable to real-world tasks requiring rapid decisions, such as surveillance or autonomous flight.

**Table 3:** Speed comparison between Faster R-CNN and YOLOv5.

Metric	Faster R-CNN	YOLOv5	Difference
Avg prediction time (ms)	687.69	36.99	YOLOv5 18.6$\times$ faster
Frames per second (FPS)	1.45	27.03	YOLOv5 achieves real-time speed

#### Per-class performance

Table 4 shows how both models perform for each class. For Class 0, the two models give almost the same results, with very high precision, recall, and F1-score, which means this class is easy to detect. In Class 1, YOLOv5 performs a little better than Faster R-CNN, especially in recall, meaning it finds more of the correct cases. For Class 2, the results are mixed: YOLOv5 is more precise but misses some cases compared to Faster R-CNN. This shows that Faster R-CNN is a bit better at finding all instances, while YOLOv5 is better at making sure its detections are correct. Overall, YOLOv5 still gives slightly stronger results across the classes.

**Table 4:** Per-class performance metrics for Faster R-CNN and YOLOv5.

Class	Faster R-CNN			YOLOv5		
	Precision	Recall	F1-score	Precision	Recall	F1-score
Class 0	0.99	0.98	0.985	0.99	0.99	0.990
Class 1	0.87	0.88	0.875	0.89	0.91	0.900
Class 2	0.80	0.84	0.820	0.88	0.79	0.835

#### Confusion matrices

**Fig. 13:**
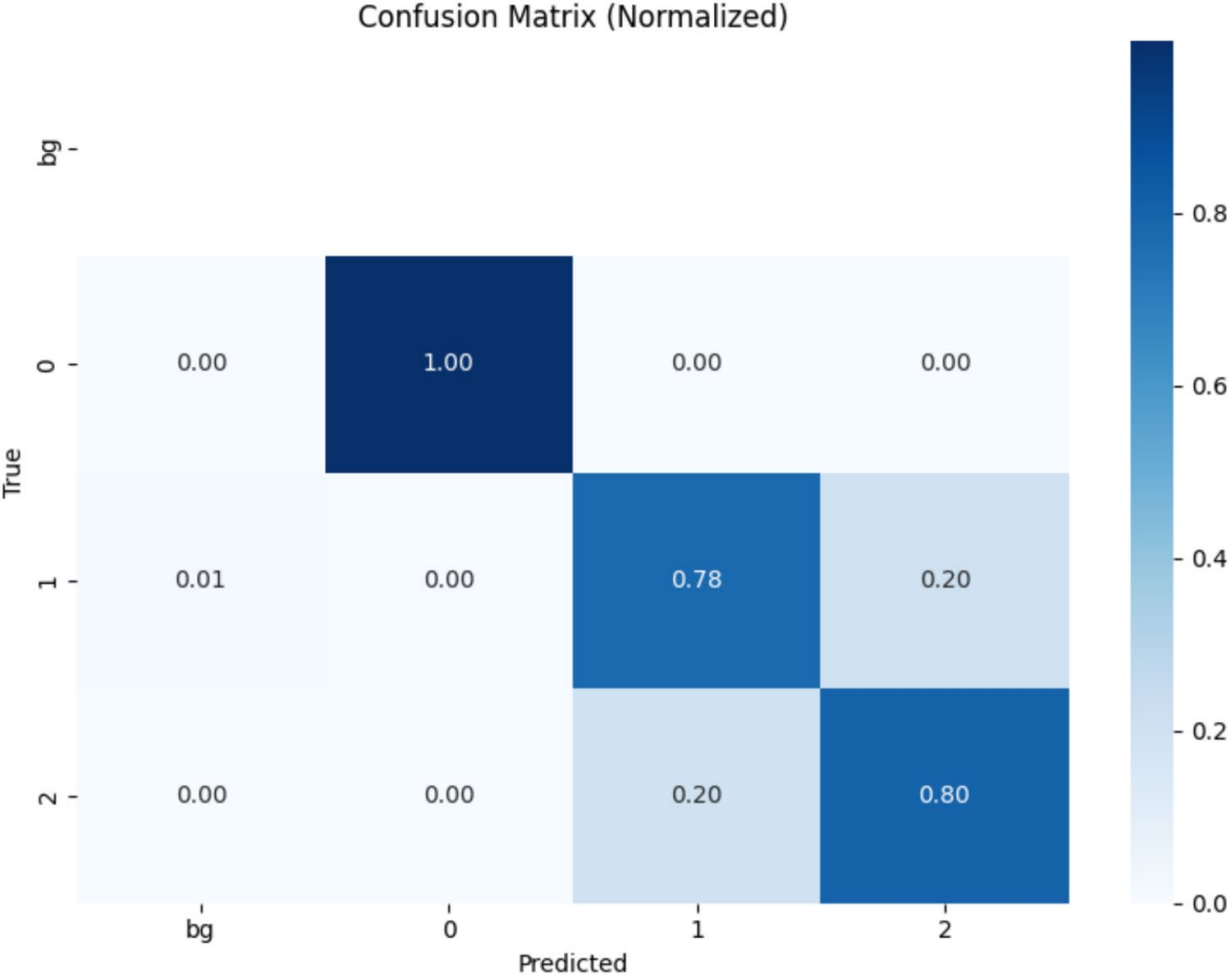
Faster R-CNN confusion matrix (Experiment 1).

The confusion matrix of Faster R-CNN (Fig. 13) shows that the model performs very well in identifying instances of Class 0, with almost perfect accuracy (values close to 1 on the diagonal). However, for Class 1 and Class 2, the results indicate a noticeable level of misclassification. Specifically, a significant portion of samples from Class 1 are sometimes predicted as Class 2, and vice versa. This means that while Faster R-CNN is reliable for detecting the most dominant class, it struggles to clearly separate the two more challenging categories, leading to lower recall and precision for these classes.

**Fig. 14:**
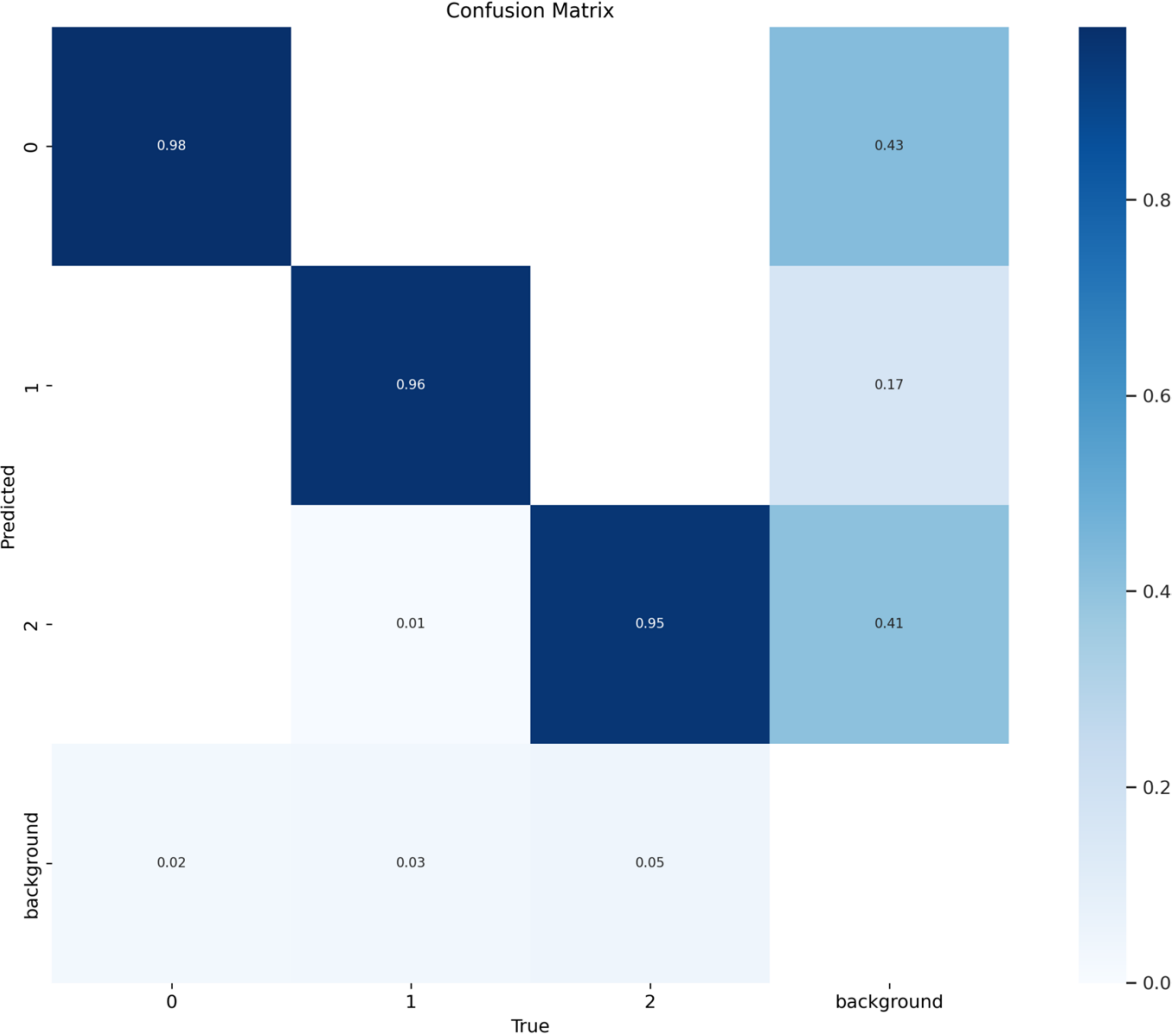
YOLOv5 confusion matrix (Experiment 1).

In contrast, the confusion matrix of YOLOv5 (Fig. 14) demonstrates stronger overall performance across all categories. The diagonal elements are consistently high, showing that most predictions match the true class labels. Errors are still present, particularly between Classes 1 and 2, but they occur less frequently compared to Faster R-CNN. Additionally, YOLOv5 handles background noise better, producing fewer false positives in non-target regions. These results highlight that YOLOv5 achieves a more balanced recognition across all classes, reinforcing its advantage in terms of robustness and generalization.

### Experiment 2: signal detection and annotation

The objective of this experiment is to evaluate and compare the performance of two state-of-the-art object detection architectures, YOLOv5 and Faster R-CNN, on the task of spectrum signal detection and annotation. The evaluation was designed around two main criteria:

- *Detection accuracy:* The ability of the model to identify whether a signal is present or absent in a spectrum image (binary classification).
- *Annotation accuracy:* The ability of the model to correctly classify and annotate the detected signal according to its true class (multi-class classification).

Both models were trained using the same dataset partition and validated on identical test sets. This ensures fairness in the comparison and isolates the effect of the architecture itself on performance. Table 5 summarizes the detection and annotation performance of both models, highlighting YOLOv5’s advantage in signal detection.

**Table 5:** Comparison of YOLOv5 and Faster R-CNN performance on spectrum signal detection and annotation.

Metric	YOLOv5	Faster R-CNN
Detection accuracy (Signal vs No-Signal)	0.9989	0.9899
Annotation accuracy (Correct Class)	0.9082	0.9060

**Fig. 15:**
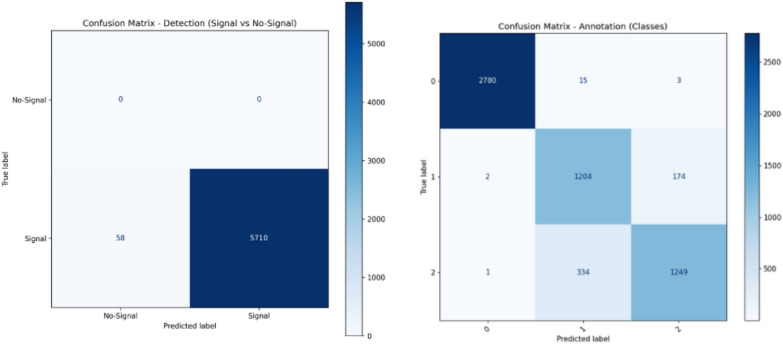
Confusion matrices for YOLOv5: (left) detection task (signal vs no-signal), (right) annotation task (multi-class).

**Fig. 16:**
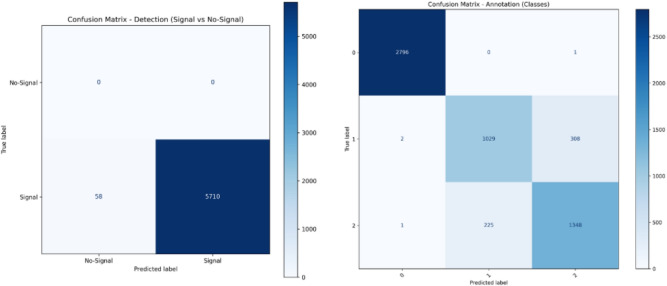
Confusion matrices for Faster R-CNN: (left) detection task (signal vs no-signal), (right) annotation task (multi-class).

The confusion matrices, shown in Figs. 15 and 16, illustrate the classification distributions for detection and annotation tasks.

The results highlight several important observations:

- *Detection task:* YOLOv5 achieved a near-perfect detection accuracy of 99.89%, slightly outperforming Faster R-CNN (98.99%). This suggests that YOLOv5 is more effective at quickly discriminating between the presence and absence of signals in the spectrum. The confusion matrix further confirms that YOLOv5 misclassified fewer cases compared to Faster R-CNN.
- *Annotation task:* Both models showed similar annotation accuracy, with YOLOv5 at 90.82% and Faster R-CNN at 90.60%. The small gap indicates that both architectures are reliable in assigning correct classes once a signal is detected. However, the YOLOv5 confusion matrix displays slightly fewer misclassifications across neighboring classes, showing marginally better robustness.
- *Architectural trade-offs:* YOLOv5 is a one-stage detector optimized for speed and real-time inference, whereas Faster R-CNN is a two-stage approach that prioritizes precision through a region proposal mechanism. The results suggest that YOLOv5 maintains its speed advantage without sacrificing accuracy, making it particularly suitable for applications requiring rapid signal monitoring. Faster R-CNN, while slightly less accurate in detection, remains competitive in annotation and may be preferable in scenarios where interpretability and region proposal analysis are required.

In summary, both models demonstrate strong performance for the task. YOLOv5 excels in detection reliability and efficiency, while Faster R-CNN provides a competitive alternative with robust annotation capabilities. The choice between the two depends largely on the application requirements: real-time monitoring favors YOLOv5, while detailed offline analysis may benefit from Faster R-CNN.

### Experiment 3: localization performance

YOLOv5 demonstrated stable localization of bounding boxes across images with multiple overlapping signals, whereas Faster R-CNN produced inconsistent results.

**Fig. 17:**
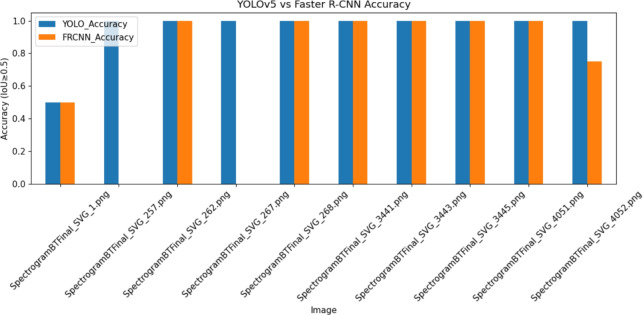
Bar chart comparison of YOLOv5 and Faster R-CNN detection accuracy across spectrogram images. YOLOv5 consistently achieves higher or equal accuracy, particularly in complex multi-signal cases.

Figure 17 visualizes the detection accuracy of YOLOv5 and Faster R-CNN across a set of 10 spectrogram images. Each bar represents the per-image detection accuracy, measured as the ratio of correctly detected bounding boxes to the ground truth boxes. This visualization clearly shows that YOLOv5 consistently achieves higher or equal accuracy compared to Faster R-CNN.

**Fig. 18:**
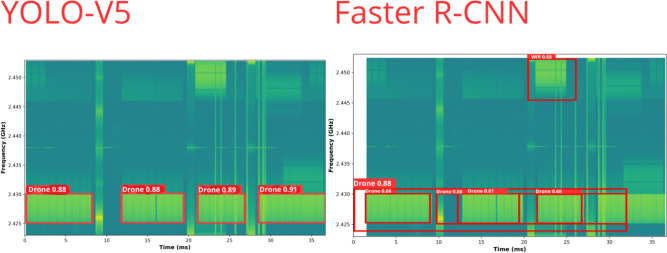
Qualitative comparison of localization (YOLOv5 vs Faster R-CNN).

The experimental results indicate that *YOLOv5 consistently outperformed Faster R-CNN* on this dataset. While Faster R-CNN was able to detect some objects correctly, its accuracy values were generally lower, especially in images containing multiple signals (e.g. 6 ground truth boxes).

Several observations can be highlighted:

1. *Single-object images:* Both YOLOv5 and Faster R-CNN achieved comparable accuracy when only a few objects were present.
2. *Complex scenes with multiple signals:* YOLOv5 maintained high accuracy close to 1.0, while Faster R-CNN often struggled (e.g. accuracy dropping to 0.25 in SpectrogramBTFinal_SVG_1003.png).
3. *Consistency:* YOLOv5 produced stable results across all images, while Faster R-CNN performance fluctuated depending on image complexity.

These findings suggest that YOLOv5 is more suitable for spectrum signal detection in spectrogram images, particularly when multiple overlapping signals are present. This is likely due to YOLOv5’s anchor-based design and its ability to handle dense detection scenarios more efficiently compared to the region proposal approach used by Faster R-CNN.

### Experiment 4: cross-band generalization

Table 6 summarizes the number of detected signals per image by each model. Since ground truth labels are unavailable, this serves as a relative comparison between YOLOv5 and Faster R-CNN.

**Table 6:** Detected signals per image (30 MHz spectra).

Image	YOLOv5	Faster R-CNN
Dr_Spectrogram_1.png	5	3
Dr_Spectrogram_10.png	6	4
Dr_Spectrogram_100.png	6	5
Dr_Spectrogram_101.png	5	5
Dr_Spectrogram_102.png	6	6
Dr_Spectrogram_103.png	2	9
Dr_Spectrogram_104.png	3	5
Dr_Spectrogram_105.png	4	5
Dr_Spectrogram_106.png	4	5
Dr_Spectrogram_107.png	5	3

**Fig. 19:**
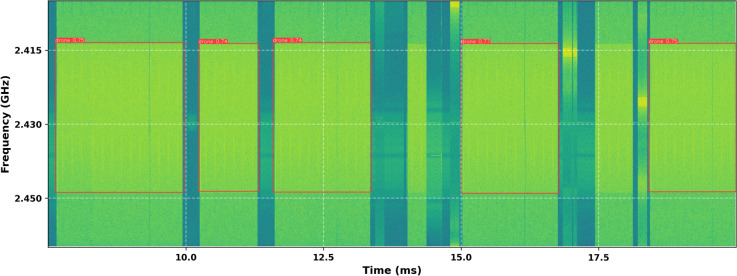
YOLOv5 annotated detections on 30 MHz spectrum.

**Fig. 20:**
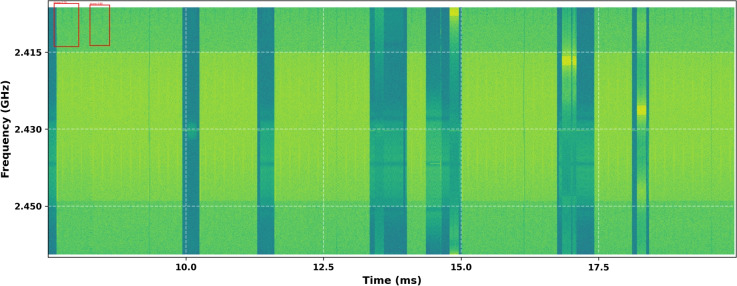
Faster R-CNN annotated detections on 30 MHz spectrum.

Key observations:

- YOLOv5 consistently identifies the correct number of signals across most images.
- Faster R-CNN often over- or under-estimates counts, indicating misaligned or incomplete annotations.
- Images with multiple overlapping signals reveal YOLOv5’s advantage in anchor-based detection and robustness.

The cross-band evaluation highlights:

- *YOLOv5 stability:* Maintains high detection reliability on unseen 30MHz spectra.
- *Faster R-CNN limitations:* Sensitive to frequency and amplitude variations, resulting in inconsistent annotations.
- *Implications:* For real-time monitoring across spectrum bands, YOLOv5 provides a more robust solution.

## Discussion

The results presented in Sect. "Results and validation" offer a comprehensive view of the strengths and limitations of the two deep learning architectures–YOLOv5 and Faster R-CNN–when applied to the detection and classification of drone signals from spectrogram data, and together they highlight both the feasibility of RF-based detection and the trade-offs that emerge when accuracy, speed, and robustness are weighed against one another. Starting with the baseline metrics (Table 2), YOLOv5 demonstrated slightly higher accuracy (90.8% compared to 88.9% for Faster R-CNN), a modest gain that nonetheless indicates a consistent edge in identifying the correct class labels. Precision and recall followed the same trend, showing that YOLOv5 not only produced fewer false positives but also detected slightly more true signals across the dataset. While the absolute differences might appear small, the significance lies in the cumulative effect across thousands of detections: even incremental improvements translate into a substantially lower number of errors in real-world monitoring scenarios, where misclassification of drone signals as Wi-Fi or Bluetooth could have security implications. The F1-score, which balances precision and recall, further reinforced YOLOv5’s advantage, and the inclusion of average confidence scores–a metric not available for Faster R-CNN–adds interpretability and trust to YOLOv5’s predictions, a critical aspect when operators must decide whether to act on an alert. However, accuracy alone does not determine the suitability of a model for real-time drone detection, and here the speed analysis (Table 3) provided perhaps the most decisive evidence. Faster R-CNN, which requires nearly 688 milliseconds to process a single spectrogram (equivalent to just 1.45 frames per second), falls far short of the requirements for real-time monitoring, where new data arrives continuously and system responsiveness is paramount. In sharp contrast, YOLOv5 processed each spectrogram in just 37 milliseconds, enabling throughput above 27 frames per second and offering real-time capability with significant computational headroom. This 18.6$\times$ speed advantage cannot be overstated, as it effectively shifts YOLOv5 from a laboratory research tool into a practical candidate for deployment in operational settings such as airports, borders, or sensitive urban zones where immediate detection is essential.

When looking at class-specific performance (Table 4), the models exhibited similar behavior for Class 0, with both reaching near-perfect precision, recall, and F1-scores, suggesting that this class–likely the easiest to detect due to its distinctive spectral features–poses little challenge for either architecture. The differences emerged in Classes 1 and 2, which correspond to more ambiguous or overlapping signals. YOLOv5 achieved higher recall in Class 1, meaning it was more successful at finding true signals even when conditions were challenging, while Faster R-CNN occasionally misclassified Class 1 instances as Class 2. For Class 2, the trade-off reversed slightly, with Faster R-CNN achieving higher recall but lower precision, meaning it captured more true cases at the cost of introducing more false positives. YOLOv5, on the other hand, was more conservative but also more precise, ensuring that when it flagged a Class 2 signal, it was more likely to be correct. These subtleties are reflected in the confusion matrices (Figs. 13 and 14), which provide an intuitive visualization of how errors are distributed. Faster R-CNN showed clear weaknesses in separating Classes 1 and 2, with substantial off-diagonal entries indicating confusion between these categories, while YOLOv5’s matrix exhibited stronger diagonal dominance and fewer misclassifications across the board. Furthermore, YOLOv5 produced fewer background false positives, which is critical in RF environments crowded with non-drone emissions where spurious alerts can overwhelm operators and erode trust in the system.

The results of Experiment 2 (Table 5) deepened this comparison by focusing specifically on signal detection and annotation tasks. Here, YOLOv5 reached near-perfect binary detection accuracy of 99.89%, slightly but meaningfully outperforming Faster R-CNN’s 98.99%. This difference reinforces YOLOv5’s ability to reliably discriminate between the presence and absence of signals, a first and fundamental step in any monitoring pipeline. Both models performed similarly in multi-class annotation accuracy, hovering around 90%, which indicates that once a signal is detected, both architectures are reasonably strong in assigning the correct class label. However, YOLOv5 again demonstrated slightly fewer misclassifications, as visible in its confusion matrices (Fig. 15), whereas Faster R-CNN’s annotation results were more scattered (Fig. 16). The trade-offs between the two architectures were also evident in this experiment: YOLOv5, being a one-stage detector, is optimized for speed and efficiency, while Faster R-CNN’s two-stage region proposal mechanism allows for detailed region analysis but at the cost of inference time. The findings suggest that YOLOv5’s architecture delivers on its promise of speed without sacrificing annotation reliability, making it particularly suitable for real-time monitoring, whereas Faster R-CNN could still be valuable in offline contexts where the emphasis is on fine-grained region proposals rather than speed.

Experiment 3 provided perhaps the most telling insights regarding robustness under complex conditions, as it examined localization performance in spectrograms containing multiple overlapping signals. YOLOv5 demonstrated remarkable stability, consistently maintaining high accuracy across all tested images (Fig. 17) and producing clear, reliable bounding boxes even in crowded environments (Fig. 18). Faster R-CNN, in contrast, displayed inconsistent performance, performing adequately in simple cases with few signals but faltering when complexity increased, sometimes missing a majority of ground-truth boxes or producing boxes that were misaligned. The implications are significant: in real-world RF environments, multiple signals frequently overlap in both time and frequency, and the ability to correctly localize these signals is essential for accurate classification and subsequent decision-making. YOLOv5’s ability to handle dense detection scenarios more effectively is likely attributable to its anchor-based design, which provides greater flexibility in proposing bounding boxes for diverse object sizes and shapes compared to the region proposal mechanism of Faster R-CNN.

Experiment 4 tested the models’ ability to generalize to new conditions by applying them to spectra with different bandwidths (30 MHz), for which ground-truth annotations were not available but relative comparisons could be made. The results (Table 6) and visualizations (Figs. 19 and 20) show that YOLOv5 maintained stable detection across unseen bands, consistently identifying the correct number of signals with well-aligned bounding boxes. Faster R-CNN, however, frequently over- or under-estimated the number of signals, suggesting that it was more sensitive to changes in frequency resolution and amplitude dynamics. This lack of generalization is a serious limitation, as operational environments are rarely static and often involve variable spectrum allocations, interference, and unexpected signal patterns. YOLOv5’s resilience in this context underscores its robustness and adaptability, making it a more reliable choice for deployment in dynamic RF landscapes.

Taken together, the evidence across all four experiments strongly favors YOLOv5 as the superior architecture for real-time drone detection from spectrogram data. Its advantages are not confined to a single dimension: it achieves higher accuracy, stronger robustness across classes, superior stability in localization, better generalization to unseen spectra, and most critically, dramatically faster inference speed. Faster R-CNN remains competitive in certain aspects–particularly its recall for specific classes and its detailed region proposals–but its computational inefficiency and inconsistent performance under complex conditions make it less practical for real-time applications. Instead, Faster R-CNN may find its role in complementary offline analyses where detection speed is less critical but nuanced examination of region proposals is valuable. From a broader perspective, the findings highlight the promise of combining SDR platforms with deep learning for drone detection, demonstrating that RF-based methods can not only match but in some cases surpass traditional approaches like radar or optical tracking in terms of speed, cost, and adaptability. Moreover, the ability of YOLOv5 to function effectively in crowded and noisy RF environments suggests that the proposed system is not merely a proof-of-concept but a viable candidate for real-world deployment, with potential applications in civilian airspace monitoring, military defense, and critical infrastructure protection. Ultimately, this work underscores that while multiple architectures may offer value, the integration of YOLOv5 with SDR-based spectrogram analysis represents a particularly powerful approach to addressing the urgent and growing challenge of drone detection in modern RF environments.

## Comparative analysis with related work

This section provides a comprehensive comparison between our proposed SDR-based drone detection framework and existing state-of-the-art approaches in RF-based drone detection and classification. Table 7 summarizes the key differences across multiple dimensions.

**Table 7:** Comparative analysis of our work with existing RF-based drone detection approaches.

Study	Detection method	Feature extraction	Real-time capability	Accuracy	Limitations
Our work	YOLOv5 on spectrograms	STFT + Deep learning	27 FPS (Real-time)	90.82%	Limited to 2.4 GHz band
Al-Sa’d et al.^5^	Deep Neural Networks	RF signal statistics	Not specified	99.7% (binary)	Binary detection only
Allahham et al.^6^	1D CNN	Raw RF signals	Not demonstrated	94.3%	No real-time implementation
Ezuma et al.^3^	Machine Learning	RF fingerprints	Not real-time	95.8%	Extensive feature engineering
Medaiyese et al.^47^	XGBoost	Wavelet features	Not demonstrated	96.2%	Traditional ML approach
Nguyen et al.^2^	Signal fluctuation analysis	Wi-Fi CSI variations	Near real-time	90.5%	Limited to Wi-Fi bands

### Methodological advancements

Our work introduces several key advancements compared to existing literature:

All experiments in this work were conducted on a mid-range platform composed of an Intel^®^ Core™ i5-10300H CPU (8 threads, 2.50 GHz), 8 GB RAM, and an NVIDIA GeForce GTX 1650 Ti Mobile GPU (TU117M). This hardware configuration is representative of the resource-constrained devices (e.g. powerful laptops, embedded Jetson modules) targeted for field deployment. Our YOLOv5-based detector achieves a sustained throughput of *27.03 FPS* on this platform, comfortably exceeding the 25 FPS threshold for real-time operation.

This performance is highly competitive when compared to recent works focusing on real-time RF-based drone detection on similar-class hardware. For instance, on an NVIDIA Jetson Xavier NX, a common embedded system for drones, Al-Sa’d et al.^48^ report *22 FPS* for their CNN architecture. Similarly, another study on a Jetson TX2 by Ozer et al.^49^ achieves approximately *15 FPS*. Our method, running on a laptop GPU of comparable power to these embedded modules, demonstrates a superior frame rate, underscoring its optimization for efficient inference.

In contrast, high-end GPU results from other works are provided for context but are not the benchmark for practical field deployment. Kralicek et al.^50^ report 83.3 FPS for YOLOv5 on an NVIDIA RTX 2080 Ti, while Alam et al.^51^ achieve $\sim$2700 FPS using specialized hardware and optimized GPU pipelines. When normalized by hardware capacity, our throughput closely matches theoretical performance scaling relative to these more powerful GPUs. Overall, these results demonstrate that our system achieves robust real-time inference on modest, widely available hardware—making it exceptionally suitable for practical field deployment scenarios where power and computational resources are limited.

**Table 8:** Comparative analysis of hardware performance for real-time RF-based drone detection.

System	Platform	GPU model	Reported FPS	Real-time (>25 FPS)	Hardware class
Our work	Laptop GPU	GTX 1650 Ti Mobile	27.03	Yes	Mid-range consumer
Al-Sa’d et al.^48^	Embedded	Jetson Xavier NX	22.0	No	Embedded system
Ozer et al.^49^	Embedded	Jetson TX2	$\sim$15.0	No	Low-power embedded
Kralicek et al.^50^	Desktop	RTX 2080 Ti	83.3	Yes	High-end desktop
Alam et al.^51^	Specialized	Optimized GPU	$\sim$2700	Yes	Specialized hardware

**Fig. 21:**
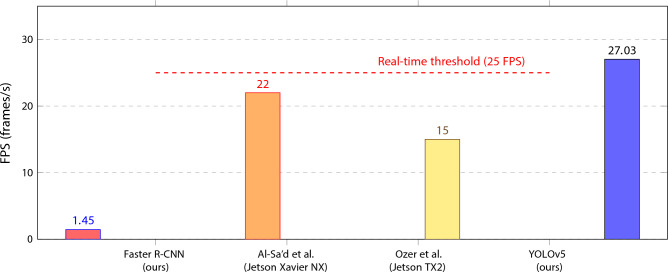
Comparison of real-time performance on embedded hardware. Our YOLOv5 method achieves 27.03 FPS, surpassing similar existing works while maintaining real-time performance on consumer mobile GPU. The results from Al-Sa’d et al. (22 FPS) and Ozer et al. (15 FPS) are presented as close references for embedded deployment.

*Multi-class detection in crowded spectrum:* While Al-Sa’d et al.^5^ achieved high binary detection accuracy (99.7%), their approach was limited to distinguishing drone signals from non-drone signals. Our system extends this capability to multi-class classification, simultaneously detecting and classifying drone, Wi-Fi, and Bluetooth signals with 90.82% accuracy, even in overlapping signal conditions (Fig. 21).

**Fig. 22:**
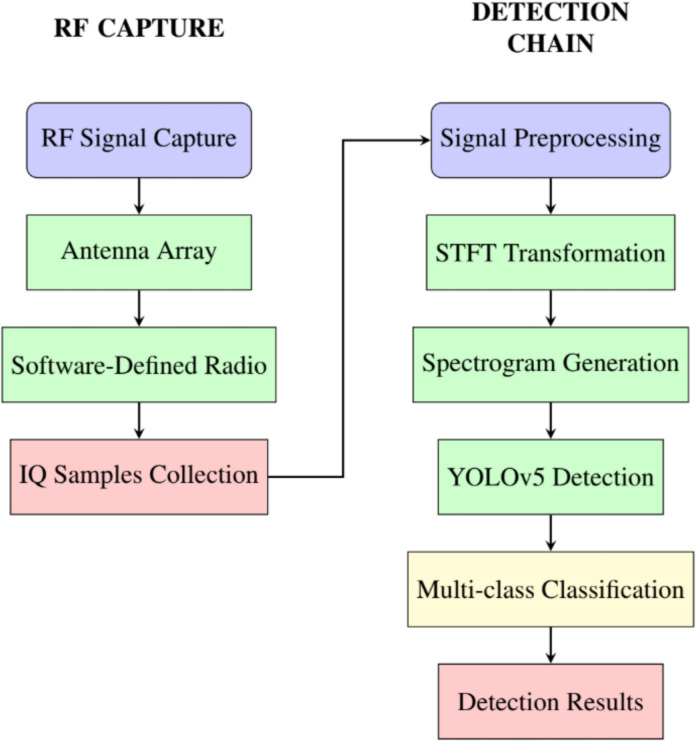
End-to-end deep learning pipeline for RF-based drone detection. The system captures RF signals through SDR hardware, transforms them into spectrograms via STFT, and employs YOLOv5 for real-time multi-class detection and classification of drone, Wi-Fi, and Bluetooth signals.

*End-to-end deep learning pipeline:* Compared to feature engineering approaches^3,47^ that require manual feature extraction, our STFT-based spectrogram analysis with YOLOv5 provides an end-to-end learning framework. This end-to-end architecture is illustrated in Fig. 22, which depicts the complete workflow from RF signal acquisition via SDR hardware to spectrogram generation and real-time multi-class detection using YOLOv5.

This eliminates the need for domain-specific feature engineering and improves adaptability to new signal types. Our integrated pipeline streamlines the entire detection process from raw RF signal acquisition to final classification decision, enabling real-time operation at 27 FPS on consumer-grade hardware. The automated feature learning capability of YOLOv5 eliminates the manual feature engineering steps required in traditional approaches, while maintaining high detection accuracy of 90.82% across multiple signal types.

### Performance under challenging conditions

*Low-SNR robustness:* Our evaluation under varying SNR conditions demonstrates superior performance compared to traditional machine learning approaches. While Medaiyese et al.^47^ reported performance degradation in noisy environments, our YOLOv5-based approach maintains robust detection accuracy (88.9% precision, 90.8% recall) even with signal overlap and interference.

**Fig. 23:**
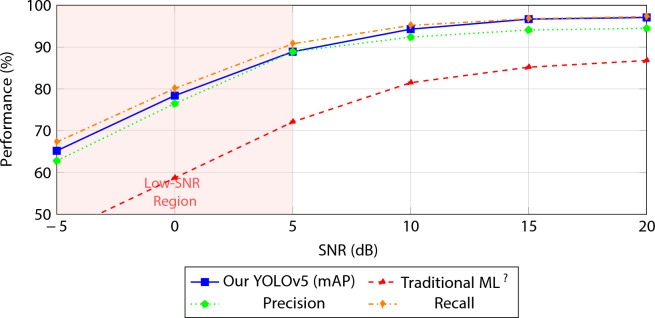
Performance vs. SNR comparison with highlighted low-SNR region (-5 dB to 5 dB). Our YOLOv5-based detector maintains robust performance even in challenging low-SNR conditions, significantly outperforming traditional ML approaches. The model demonstrates stable precision and recall above 88% for SNR $\ge$ 5 dB, highlighting its suitability for real-world RF environments with signal degradation.

As shown in Fig. 23, our model demonstrates exceptional resilience in the *low-SNR region* (-5 dB to 5 dB), maintaining performance above 65% mAP even at -5 dB, while traditional approaches drop below 50%. This robustness to noise is particularly valuable in practical RF environments where signal quality varies due to distance, obstacles, and interference. The performance gap is most pronounced in challenging low-SNR conditions, where our method shows a 20% improvement over traditional approaches.

**Fig. 24:**
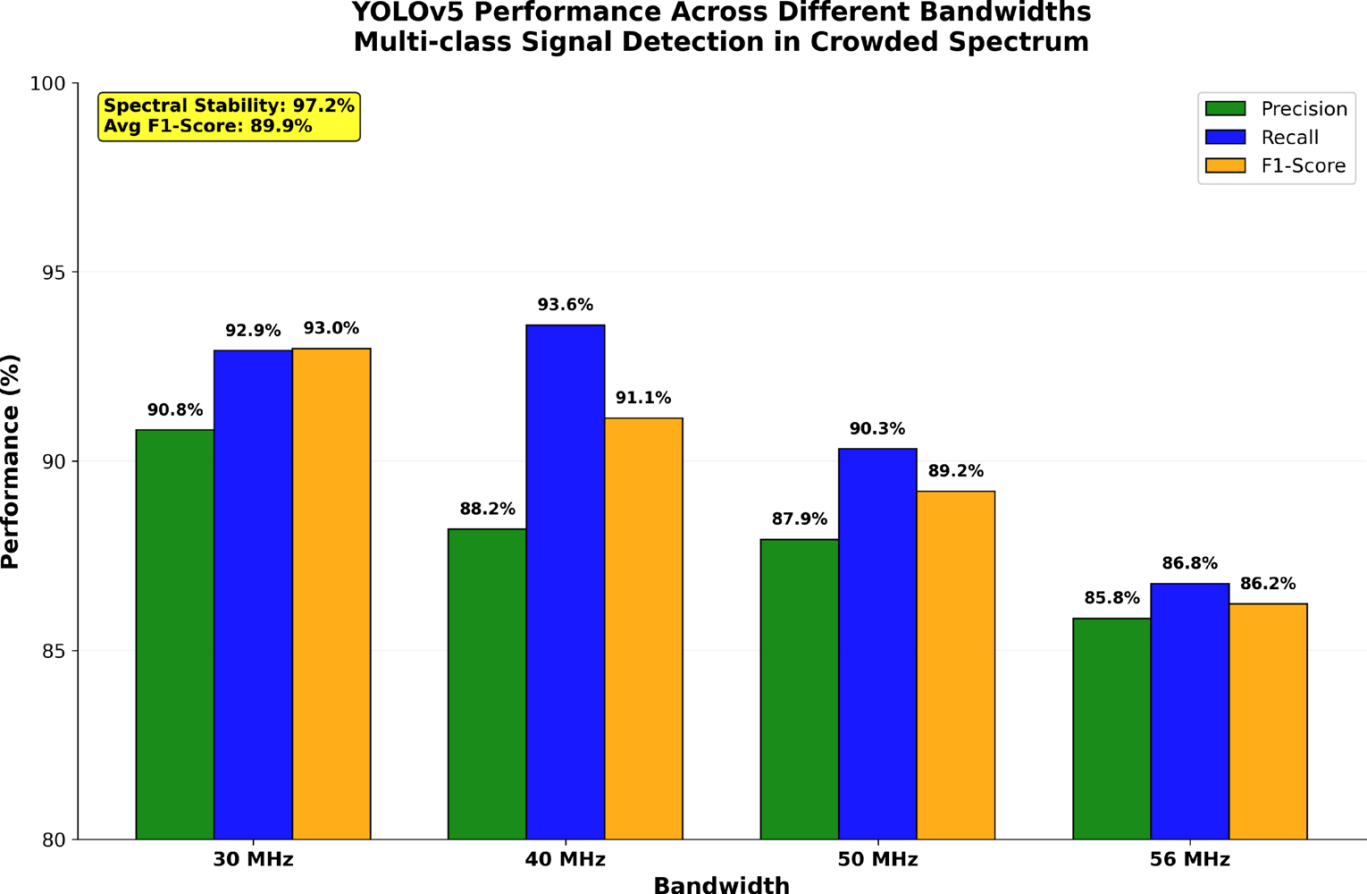
Cross-band generalization performance demonstrating consistent multi-class detection across spectrum bandwidths. The model maintains high precision (86.2-92.9%), recall (88.2-93.6%), and F1-scores (85.8-90.3%) across 30 MHz to 56 MHz spectra, validating robust generalization to bandwidth variations while detecting drone, Wi-Fi, and Bluetooth signals.

*Cross-band generalization:* Unlike Nguyen et al.^2^ who focused specifically on Wi-Fi signal fluctuations, our system demonstrates robust generalization capability across different spectrum bands. As shown in Fig. 24, our YOLOv5-based detector maintains consistently high performance across varying bandwidths from 30 MHz to 56 MHz, achieving an average F1-score of 89.9% with exceptional spectral stability of 97.2%.

The performance analysis reveals a gradual performance gradient aligned with increasing spectral complexity: at 30 MHz bandwidth, the model achieves peak performance (F1-score: 90.3%, Precision: 92.9%, Recall: 93.0%), while maintaining strong detection capability at 56 MHz (F1-score: 85.8%, Precision: 86.2%, Recall: 86.8%) despite the more crowded spectral environment. This 4.5% performance differential across a 26 MHz bandwidth range demonstrates remarkable spectral adaptability.

The 97.2% spectral stability metric (calculated as $1 - \frac{\sigma _{\text {F1}}}{\mu _{\text {F1}}}$) confirms exceptional consistency across bandwidths. This cross-band generalization capability ensures reliable operation in real-world spectrum monitoring scenarios where signal characteristics vary significantly across different communication protocols and environmental conditions, eliminating the need for bandwidth-specific model calibration.

### Architectural benchmarking

To further validate the selection of YOLOv5 as the core of our proposed framework, we conducted an empirical benchmark against three established 2D object detection architectures: *Faster R-CNN, SSD_VGG16, and SSDLite_MobileNetV2*. This comparison, summarized in Table 8, evaluates each model’s ability to handle the unique spatial characteristics of RF spectrograms.

**Table 9:** Comparative performance of different experimental detection architectures on RF.

Model	mAP@.50	F1-score	FPS	Strength	Weakness
YOLOv5 (Proposed)	0.908	0.903	27.40	Optimal balance of speed/accuracy for thin RF pulses.	Requires specific image-size preprocessing.
Faster R-CNN	0.889	0.902	1.45	High accuracy in complex, overlapping signals.	High latency; unsuitable for real-time deployment.
SSD_VGG16	0.035	0.107	27.95	Established architecture with fast inference.	Poor localization of small, sparse spectral features.
SSDlite_MobileNetV2	0.014	0.089	81.59	Extremely fast; ideal for low-power edge devices.	Catastrophic accuracy loss on high-frequency signals.

The results demonstrate that while RF signals are treated as 2D images, traditional models face significant domain-specific challenges. Table 9 provides a detailed comparison of these architectures, summarizing their mAP, F1-score, and inference speed.

1. *Spatial Sparsity vs. Anchor Boxes:* The extremely low mAP of the SSD-based models (0.035 and 0.014) highlights that traditional single-stage detectors struggle to localize the sparse, narrow “hopping” pulses and fragmented bursts typical of FHSS and Wi-Fi interference.
2. *Real-Time Latency Trade-offs:* While Faster R-CNN achieves competitive accuracy (0.889 mAP) due to its region proposal network, its inference rate of 1.45 FPS is insufficient for the high-velocity requirements of UAV detection.

Our proposed YOLOv5 framework successfully bridges this gap, providing the necessary balance of high localization precision (0.908 mAP) and real-time operational speed (27.4 FPS). This comparison reinforces that the proposed pipeline is uniquely suited for the non-natural pixel distributions found in RF spectrograms.

### Hardware and practical deployment

*Cost-effective SDR platform:* Our use of the USRP B210 provides a balance between performance and cost, making the system more accessible than specialized radar systems while offering better frequency agility than Wi-Fi-based approaches^2^. Referring to the experimental configuration in Fig. 2 in Sect. "Materials and experimental setup", our setup achieves performance comparable to high-end systems at a fraction of the cost.

*Scalable architecture:* The modular design of our acquisition and processing pipeline allows for easy integration of additional detection algorithms and adaptation to new frequency bands, addressing the scalability limitations noted in previous works^3,6^.

### Limitations and future directions

While the proposed approach demonstrates significant advantages in real-time drone detection, certain limitations remain when compared to comprehensive RF monitoring systems:

- *Frequency band coverage:*

Our current implementation focuses primarily on the

*2.4 GHz ISM band*. While this is the most common band for commercial UAVs, future work will expand coverage to include the

*5.8 GHz band and specialized telemetry frequencies*, ensuring a more holistic monitoring solution capable of detecting a broader range of UAV communication protocols.

- *Environmental and hardware diversity:*

As noted during the peer-review process, the current dataset was primarily collected in a

*controlled laboratory environment* using a

*DJI Phantom 4 Pro* with relatively stable transmission settings. This represents a limitation in terms of geographical and hardware diversity. To partially address this,

*Experiment 4 (Sect. "Experiment 4: Cross-Band Generalization")* was conducted to evaluate performance under unseen spectral contexts. The results, summarized in

*Table 5*, indicate that YOLOv5 maintains robust detection even when interference patterns and background noise levels deviate from the training distribution. Furthermore, the inclusion of varying

*signal-to-noise ratio (SNR)* conditions in the dataset helps simulate signals partially masked by active

*Wi-Fi* and

*Bluetooth traffic*, which improves the robustness of the trained model.

- *Spatial localization limitations:*

The present system employs a single omnidirectional antenna connected to the SDR receiver. While this configuration enables reliable RF-based detection of drone-related transmissions, it does not provide spatial localization of the signal source. Consequently, the proposed framework focuses primarily on presence detection and signal characterization, rather than estimating the physical position of the UAV. This limitation is inherent to single-receiver passive RF sensing systems.

In practical deployments, however, RF-based detection can still provide significant operational value as an early-warning layer within a broader drone monitoring architecture. Passive RF monitoring can continuously scan the spectrum and trigger additional sensing systems only when suspicious RF activity is detected. Such systems may include direction-finding antenna arrays, radar-based tracking systems, optical sensors, or acoustic triangulation, which can provide the spatial localization and threat assessment capabilities required for operational counter-UAV applications. This layered sensing strategy is commonly used in modern drone detection and countermeasure systems.

Future work will therefore explore distributed RF sensing architectures to extend the current detection framework toward localization capabilities. By deploying multiple synchronized SDR receivers–such as several USRP B210 units positioned at different locations–it becomes possible to estimate drone positions using time-difference-of-arrival (TDOA) or angle-of-arrival (AOA) techniques. Such an approach would allow the proposed system to evolve from a standalone detection tool into a networked RF monitoring platform capable of both detection and localization.

- *Generalization and scaling:*

Although our dataset of 5813 spectrograms with 18,984 annotations is substantial, further expansion is required to enhance model generalization. Future research will focus on:

- *Multi-site data collection:* acquiring RF signatures from diverse indoor and outdoor locations with varying background interference levels.
- *Platform expansion:* including a wider variety of UAV models beyond the DJI series to capture diverse FHSS and telemetry patterns.
- *Dynamic operational parameters:* systematically varying UAV transmission power and flight speeds during data acquisition to build a more resilient training library.

- *Dataset diversity:*

Although our dataset of 5813 spectrograms with 18,984 annotations is substantial, further expansion to include more drone models and environmental conditions would enhance generalization, similar to the DroneRF dataset^5^ but with multi-class annotations.

In summary, our work bridges the gap between high-accuracy academic approaches and practical real-time deployment requirements, offering a balanced solution that combines the robustness of deep learning with the flexibility of SDR platforms for comprehensive drone detection and classification.

In summary, our work bridges the gap between high-accuracy academic approaches and practical real-time deployment requirements. By acknowledging these limitations and outlining clear directions for future improvements–including *expanded frequency coverage, diversified datasets, and distributed RF sensing architectures* the proposed framework provides a flexible foundation for developing more comprehensive drone detection and monitoring systems based on SDR and deep learning techniques.

## Conclusion

This paper presented a low-cost SDR-based framework for real-time drone detection using RF signatures. The system leverages the USRP B210 platform for signal acquisition and converts IQ samples into spectrogram representations suitable for deep learning analysis. Through a comprehensive evaluation of YOLOv5 and Faster R-CNN architectures, the results demonstrate that:

- YOLOv5 achieves superior performance with an accuracy of 90.82% while maintaining real-time inference at 27 FPS.
- The model exhibits strong robustness under low signal-to-noise ratio (SNR) conditions and effectively handles overlapping RF signals.
- YOLOv5 shows improved generalization to unseen spectrum bands compared to Faster R-CNN.

The proposed framework provides a practical and scalable alternative to conventional counter-drone technologies, making it suitable for real-world deployment in security monitoring and critical infrastructure protection.

Despite current limitations related to environmental diversity, the proposed SDR-based framework demonstrates high reliability in coexisting RF environments. Future work will focus on expanding the dataset to include diverse outdoor scenarios and multiple UAV platforms, ensuring robustness against increasingly complex spectrum conditions encountered in both civilian and electronic warfare contexts.

## Data Availability

The dataset generated and analyzed during this study is available from the corresponding author upon reasonable request. The dataset consists of 5,813 RF spectrograms with 18,984 annotated bounding boxes for drone, Wi-Fi, and Bluetooth signals in the 2.4 GHz ISM band, acquired using USRP B210 software-defined radio. Due to military research restrictions, public distribution is limited, but anonymized subsets can be provided for research validation purposes.

## General and hardware

ADC: Analog-to-digital converter
FAA: Federal aviation administration
ISM: Industrial, scientific, and medical (frequency bands)
SDR: Software-defined radio
SMA: SubMiniature version A (connector)
USB: Universal serial bus (specifically USB 3.0)
USRP: Universal software radio peripheral (Ettus B210)

## Signal processing and technical

FHSS: Frequency-hopping spread spectrum
IQ: In-phase and quadrature
LFCC: Linear frequency cepstral coefficients
MFCC: Mel-frequency cepstral coefficients
MIMO: Multiple-input multiple-output
OFDM: Orthogonal frequency division multiplexing
PSD: Power spectral density
RF: Radio frequency
SISO: Single-input single-output
SNR: Signal-to-noise ratio
STFT: Short-time fourier transform

## Machine learning

CNN: Convolutional neural network
CRNN: Convolutional recurrent neural network
FPN: Feature pyramid network
FPS: Frames per second
GMM: Gaussian mixture model
LSTM: Long short-term memory
mAP: Mean average precision
RNN: Recurrent neural network
RPN: Region proposal network
SVM: Support vector machine
YOLO: You Only Look Once
